# Sex-specific trisomic *Dyrk1a*-related skeletal phenotypes during development in a Down syndrome model

**DOI:** 10.1242/dmm.050914

**Published:** 2024-09-23

**Authors:** Jonathan M. LaCombe, Kourtney Sloan, Jared R. Thomas, Matthew P. Blackwell, Isabella Crawford, Flannery Bishop, Joseph M. Wallace, Randall J. Roper

**Affiliations:** ^1^ Department of Biology, Indiana University Indianapolis, Indianapolis, IN 46202, USA; ^2^ Labcorp Early Development Laboratories, Inc., Greenfield, IN 46140, USA; ^3^ Department of Biomedical Engineering, Purdue University, Indianapolis, IN 46202, USA

**Keywords:** Trisomy 21, Bone, Appendicular, Development, *Dyrk1a*, Dosage imbalance

## Abstract

Skeletal insufficiency affects all individuals with Down syndrome (DS) or trisomy 21 and may alter bone strength throughout development due to a reduced period of bone formation and early attainment of peak bone mass compared to those in typically developing individuals. Appendicular skeletal deficits also appear in males before females with DS. In femurs of male Ts65Dn DS model mice, cortical deficits were pronounced throughout development, but trabecular deficits and *Dyrk1a* overexpression were transitory until postnatal day (P) 30, when there were persistent trabecular and cortical deficits and *Dyrk1a* was trending toward overexpression. Correction of DS-related skeletal deficits by a purported DYRK1A inhibitor or through genetic means beginning at P21 was not effective at P30, but germline normalization of *Dyrk1a* improved male bone structure by P36. Trabecular and cortical deficits in female Ts65Dn mice were evident at P30 but subsided by P36, typifying periodic developmental skeletal normalizations that progressed to more prominent bone deficiencies. Sex-dependent differences in skeletal deficits with a delayed impact of trisomic *Dyrk1a* are important to find temporally specific treatment periods for bone and other phenotypes associated with trisomy 21.

## Teaser

**Summary:** Analysis of the developing bone in Ts65Dn Down syndrome model mice reveals timepoints during development when trisomic *Dyrk1a* is linked to appendicular skeletal abnormalities, despite *Dyrk1a* not always being overexpressed.

## INTRODUCTION

All individuals with trisomy 21 (Ts21) have skeletal abnormalities and are at risk for osteopenia and osteoporosis. Different from typically developing individuals, both males and females with Down syndrome (DS) exhibit deficits in bone structure and bone accrual during adolescence, and males with DS decline in skeletal parameters earlier than females with DS. A reduced period of bone accrual may also make individuals with DS more susceptible to bone breakage during adolescence (de Moraes et al., 2008). People with DS attain peak bone mass 5-10 years earlier than the general population and experience bone loss sooner and at a higher rate than the general population (Carfi et al., 2017; Costa et al., 2018). Growth velocity is reduced in children with DS and skeletal age is delayed compared to chronological age. We have reported that 27% of individuals with DS reported suffering a fracture or broken bone, and most fractures occurred in individuals under 20 years of age (although most individuals that responded were <20 years old) (LaCombe and Roper, 2020). The maximal height in people with DS is reached around 15 years of age, which is precocious compared to the general population (de Moraes et al., 2008; Angelopoulou et al., 1999; Myrelid et al., 2002).

In individuals without DS, age-related trabecular bone loss begins around 30 years of age and cortical bone loss begins around 50 years of age (Almeida, 2012; Khosla, 2013; Manolagas et al., 2013). Recent comprehensive studies with large sample sizes of adults with DS have shown significant differences in bone mineral density (BMD) in individuals with DS beginning in their second and third decades of life. Males with DS begin losing BMD in the femur much earlier than females with DS (30 compared to 40 years of age, respectively), suggesting a protective effect of the female sex in terms of maintaining BMD (Carfi et al., 2017; Costa et al., 2017, 2018; Tang et al., 2019). Additionally, because the average life expectancy of individuals with DS has increased to more than 60 years of age (Baird and Sadovnick, 1989; Bittles and Glasson, 2004; Weijerman and de Winter, 2010), more individuals with DS will likely suffer from osteoporosis and fractures in older years. Therefore, there is a crucial need to address the etiology, including the impact of trisomic genes, of bone deficiencies and osteoporosis in males and females with DS.

Ts(17^16^)65Dn (Ts65Dn) mice are the most studied mouse model of DS and exhibit many DS-related phenotypes, including skeletal abnormalities (Blazek et al., 2015a,b, 2011; Reeves et al., 1995; Thomas et al., 2021). These phenotypes are attributed to the presence of triplicated genes on a freely segregating minichromosome composed of the distal arm of mouse chromosome 16 (Mmu16) attached to the centromeric region of Mmu17. In addition to increased gene dosage of ∼100 genes orthologous to human chromosome 21 (Hsa21), ∼35 protein-coding genes are also triplicated in the centromeric region of Mmu17 that are not homologous to those in Hsa21 (Duchon et al., 2011; Reinholdt et al., 2011). Most studies on Ts65Dn mice have been limited to males, primarily due to their subfertile nature, which requires the use of females for colony maintenance (Roper et al., 2006; Moore et al., 2010). Male Ts65Dn mice have femoral skeletal deficiencies at 6 weeks (a time of bone formation roughly equivalent to the skeletal age of humans under 20 years of age) and 16 weeks (a time of skeletal maturity in mice similar to humans between 20 and 30 years of age) (Blazek et al., 2015a, 2011). Histological assessment of appendicular trabecular bone in male Ts65Dn mice at 6 weeks showed a reduced bone formation rate (BFR), mineral apposition rate (MAR) and mineralizing surface, and increased osteoclast number (Blazek et al., 2015a). At 3 months (∼12 weeks), tibial BFR, the percentage of osteoblast surface to bone surface, the percentage of osteoclasts to bone surface, and osteoclast number were reduced in Ts65Dn male mice (Fowler et al., 2012). The bone formation marker P1NP was decreased significantly in Ts65Dn mice compared to euploid mice at 24 months and in humans with DS at 19-51 years compared to control individuals, whereas the bone resorption marker TRAP 5b was decreased in Ts65Dn mice at 24 months; however, the bone resorption marker CTx was not significantly decreased in humans with DS aged 19-51 years (Fowler et al., 2012; McKelvey et al., 2013). Female Ts65Dn mice at 6 weeks have also been shown to have trabecular deficits, including lower BMD and increased trabecular separation (Tb.Sp), and cortical deficits, including smaller total cortical cross-sectional area (Tt.Ar), periosteal perimeter (Ps.Pm) and endocortical perimeter (Ec.Pm), compared to female euploid mice (Thomas et al., 2021).

It has been hypothesized that *Dyrk1a*, a gene found in three copies in humans with DS and in Ts65Dn mice, significantly contributes to many DS phenotypes, including skeletal malformations (Arron et al., 2006; Duchon and Herault, 2016; Sloan et al., 2023). DYRK1A is a serine-threonine kinase that regulates many downstream proteins and transcription factors including cyclin D1 and NFAT (Arron et al., 2006; Branchi et al., 2004; Guedj et al., 2012; Park et al., 2009; Atas-Ozcan et al., 2021). Male and female *Dyrk1a* transgenic mice (increased copy number of just *Dyrk1a*) exhibited significantly reduced bone mass including decreased bone volume fraction and reduced trabecular skeletal parameters (Lee et al., 2009). The deficient skeletal phenotype of *Dyrk1a* transgenic mice was characterized by osteoblast deficiencies that resulted in the low bone mass phenotype (Lee et al., 2009). Returning *Dyrk1a* to two functional copies from conception in otherwise trisomic Ts65Dn mice (Ts65Dn,*Dyrk1a*^+/−^) rescued femoral trabecular skeletal parameters in male 6-week-old Ts65Dn mice to euploid levels, improved cortical cross-sectional area and normalized femoral trabecular and cortical MAR and BFR (Blazek et al., 2015a). Yet, normalization of *Dyrk1a* in otherwise trisomic Ts65Dn mice did not have a significant corrective role in developing skeletal abnormalities at embryonic day (E) 17.5 (Blazek et al., 2015b). These results suggest a time-dependent role of trisomic *Dyrk1a* in Ts65Dn mice and possibly in individuals with DS.

Several reports describe potential DYRK1A inhibitors and their possible use for correcting DS-related cognitive deficits (Duchon and Herault, 2016; de la Torre and Dierssen, 2012; Becker et al., 2014). Treatments using supplements containing epigallocatechin-3-gallate (EGCG) – a putative DYRK1A inhibitor – have been reported to improve cognitive performance in DS mouse models and in some measures in clinical trials (De la Torre et al., 2014, 2016; Pons-Espinal et al., 2013; Souchet et al., 2019). In contrast, no significant beneficial effects on cognitive function were found in DS model mice using pure EGCG treatments (Stringer et al., 2015, 2017a; Goodlett et al., 2020), and evidence shows that high concentrations and prolonged treatment with EGCG harms skeletal structure (Jamal et al., 2022). CX-4945 is a repurposed anti-cancer drug that displays a higher affinity for DYRK1A than harmine and INDY inhibitors (Kim et al., 2016) and was shown to be effective against hematological cancers and in reducing osteoclast activity while increasing osteoblast activity, suggesting an overall inhibitory effect on the hematopoietic progenitor lineage and a stimulatory effect on the mesenchymal lineage (Son et al., 2013; Chon et al., 2015). Because of its high affinity for DYRK1A and its impact on osteoblasts and osteoclasts, CX-4945 may be an excellent candidate to treat skeletal abnormalities associated with DS, especially at times when *Dyrk1a* is overexpressed.

We hypothesized that *Dyrk1a*-related appendicular skeletal phenotypes emerge during development between E17.5 and postnatal day (P) 42 (6 weeks) in Ts65Dn mice and overexpression of *Dyrk1a* during a crucial developmental window in the femoral compartment of Ts65Dn mice dysregulates molecular mechanisms that cause aberrant skeletal phenotypes. Similar to humans with DS, we further posited that there would be a sexual dimorphism in skeletal deficiencies found in Ts65Dn mice. Additionally, we hypothesized that this altered expression of *Dyrk1a* could be temporally corrected by genetic and therapeutic means to improve skeletal phenotypes associated with DS in mouse models. Identifying the age at which a *Dyrk1a*-related appendicular skeletal phenotype emerges is crucial to determine the genetic, molecular and cellular processes that are dysregulated to cause persistent phenotypic changes into adulthood.

## RESULTS

### Development of trabecular deficits in male Ts65Dn DS model mice from P12 to P42

**Fig. 1.**
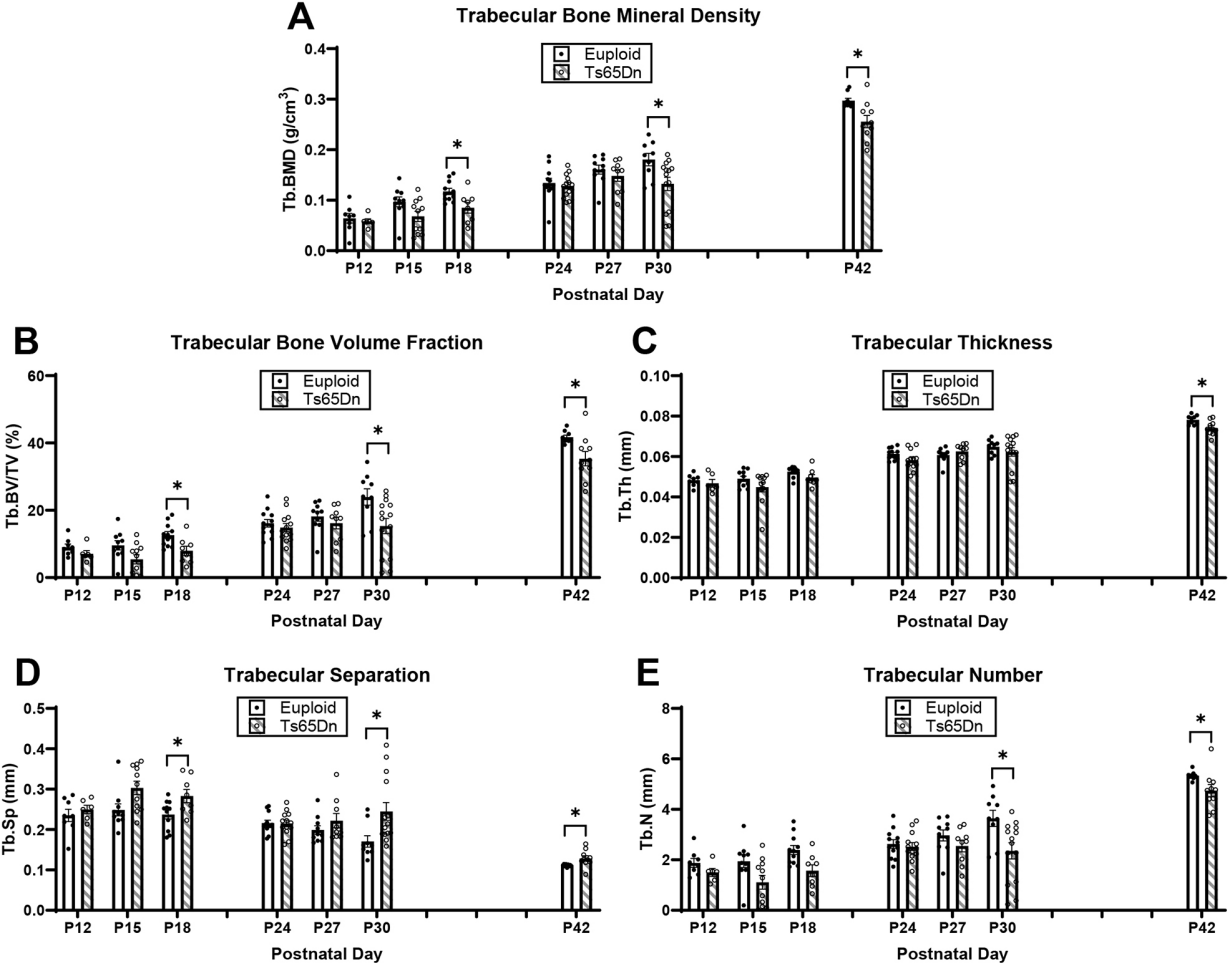
**Trabecular analysis of male Ts65Dn femurs from P12 to P42.** (A-E) Trabecular bone mineral density (Tb.BMD) (A), bone volume fraction (Tb.BV/TV) (B), thickness (Tb.Th) (C), separation (Tb.Sp) (D) and number (Tb.N) (E) were assessed using micro-computed tomography in male Ts65Dn mice and euploid littermate control mice from postnatal day (P) 12 to P42. Data are mean±s.e.m. P12, *n*=8 euploid and 6 Ts65Dn mice; P15, *n*=10 euploid and 11 Ts65Dn mice; P18, *n*=11 euploid and 8 Ts65Dn mice; P24, *n*=12 euploid and 13 Ts65Dn mice; P27, *n*=10 euploid and 9 Ts65Dn mice; P30, *n*=9 euploid and 14 Ts65Dn mice; P42, *n*=9 euploid and 10 Ts65Dn mice. Significance was determined through two-tailed unpaired *t*-test with FDR adjustment. *P≤0.05.

We have previously observed trabecular and cortical skeletal deficits in 6- and 16-week-old DS mouse models that vary across age (Blazek et al., 2011; Sloan et al., 2023; Thomas et al., 2020, 2021). To further understand the development of these skeletal deficits, we first examined trabecular and cortical bone development in male Ts65Dn DS model mice and euploid littermate control mice from P12 to P42. We detected significant genotype and age effects when trabecular bone measurements were analyzed together from P12 to P42 (Table S1). When trabecular phenotypes at each age were analyzed independently, we found that at P12 and P15, there were no significant differences between trisomic and euploid mice; at P18, Ts65Dn male mice had significantly reduced BMD (*P*=0.034) and bone volume fraction (BV/TV) (*P*=0.023) and increased Tb.Sp (*P*=0.035) (Fig. 1A-E). Additionally, trabecular deficits were not seen in trisomic male mice at P24 and P27. At P30, trabecular phenotypes characteristic of adult male Ts65Dn mice emerged, evidenced by significant reduction in BMD (*P*=0.030), BV/TV (*P*=0.030) and trabecular number (Tb.N) (*P*=0.033), and an increase in Tb.Sp (*P*=0.050). Similar to what has been reported at P42 (6 weeks), we found that P42 Ts65Dn mice had significantly reduced BMD (*P*=0.040), BV/TV (*P*=0.025), trabecular thickness (Tb.Th) (*P*=0.023) and Tb.N (*P*=0.043), and significantly increased Tb.Sp (0.036) compared to euploid mice.

### Cortical deficits in male Ts65Dn DS model mice from P12 to P42

**Fig. 2.**
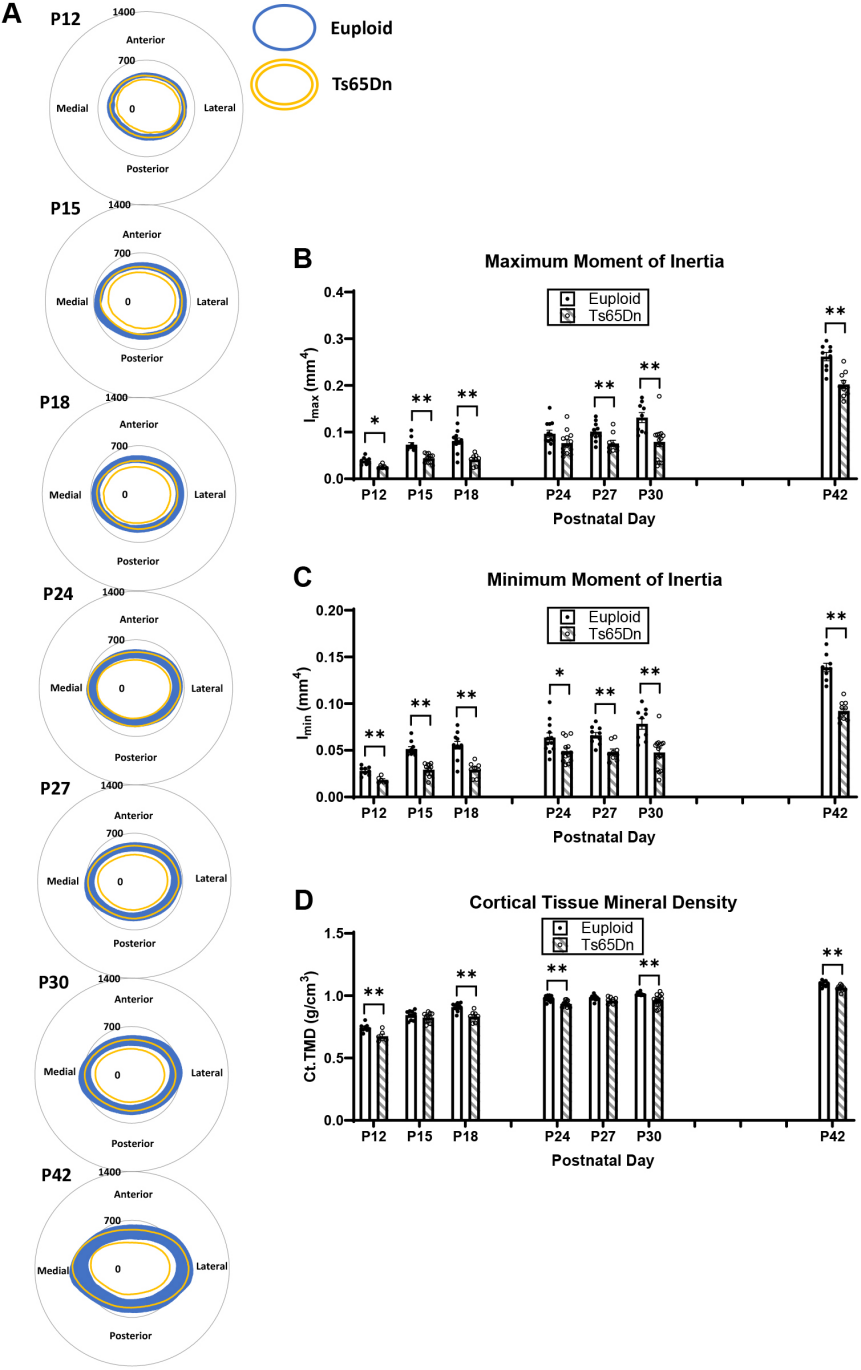
**Cortical analysis of male Ts65Dn femurs from P12 to P42.** (A) Cortical models of euploid (solid blue regions) and Ts65Dn (yellow outlined regions) were made by finding the centroid of the bone, then measuring the inner and outer radii every 0.5° in MATLAB for each cortical slice (total of seven) for each animal, and then finding the average for the entire group. Numbers indicate the radii of the grey circles in micrometers. (B-D) Maximum moment of inertia (I_max_) (B), minimum moment of inertia (I_min_) (C) and cortical tissue mineral density (Ct.TMD) were assessed using micro-computed tomography in male Ts65Dn mice and euploid littermate control mice from P12 to P42 (see Fig. S2 for other cortical parameters). Data are mean±s.e.m. See the legend of Fig. 1 for group numbers. Significance was determined through two-tailed unpaired *t*-test with FDR adjustment. *P≤0.05; **P≤0.01.

Significant genotype and age effects were found when cortical bone measures were analyzed from P12 to P42 (Table S1). Examination of specific cortical phenotypes at each age showed that at P12, there were significant cortical deficits in all cortical measures in Ts65Dn compared to those in euploid male mice: Tt.Ar (*P*=0.011), marrow area (Ma.Ar) (*P*=0.032), cortical area (Ct.Ar) (*P*=0.011), cortical thickness (Ct.Th) (*P*=0.019), Ps.Pm (*P*=0.012), Ec.Pm (*P*=0.034), maximum moment of inertia (I_max_) (*P*=0.009), minimum moment of inertia (I_min_) (*P*=0.009) and cortical tissue mineral density (Ct.TMD) (*P*=0.009) (Fig. 2A-D; Fig. S2). At P15, there was a significant reduction in all cortical measures (except Ct.TMD) in Ts65Dn compared to euploid littermate male mice: Tt.Ar (*P*=0.005), Ma.Ar, (*P*=0.005), Ct.Ar (*P*=0.005), Ct.Th (*P*=0.028), Ps.Pm (*P*=0.005), Ec.Pm (*P*=0.005), I_max_ (*P*=0.006) and I_min_ (*P*=0.006). In P18, all cortical measurements (including Ct.TMD) were significantly reduced in trisomic compared to euploid male mice: Tt.Ar (*P*=0.002), Ma.Ar (*P*=0.002), Ct.Ar (*P*=0.002), Ct.Th (*P*=0.002), Ps.Pm (*P*=0.002), Ec.Pm (*P*=0.002), I_max_ (*P*=0.002), I_min_ (*P*=0.002) and Ct.TMD (*P*=0.002). At P24, only Ct.Ar (*P*=0.027), Ct.Th (*P*=0.014), I_min_ (*P*=0.045) and Ct.TMD (0.008) were significantly reduced in trisomic compared to control male mice. At P27, cortical defects were again significantly different in Tt.Ar (*P*=0.005), Ma.Ar (*P*=0.005), Ps.Pm (*P*=0.004), Ec.Pm (*P*=0.004), I_max_ (*P*=0.023) and I_min_ (*P*=0.005). At P30, all cortical parameters were significantly reduced in trisomic compared to euploid male mice: Tt.Ar (*P*=0.005), Ma.Ar (*P*=0.004), Ct.Ar (*P*=0.004), Ct.Th (*P*=0.004), Ps.Pm (*P*=0.004), Ec.Pm (*P*=0.004), I_max_ (*P*=0.004), I_min_ (*P*=0.005) and Ct.TMD (*P*=0.005). At P42, similar to what has been previously reported (Thomas et al., 2021), all cortical measurements except Ct.Th were significantly reduced in male Ts65Dn mice compared to euploid mice: Tt.Ar (*P*=0.002), Ma.Ar (*P*=0.002), Ct.Ar (*P*=0.002), Ps.Pm (*P*=0.002), Ec.Pm (*P*=0.002), I_max_ (*P*=0.002), I_min_ (*P*=0.002) and Ct.TMD (*P*=0.005).

**Fig. 3.**
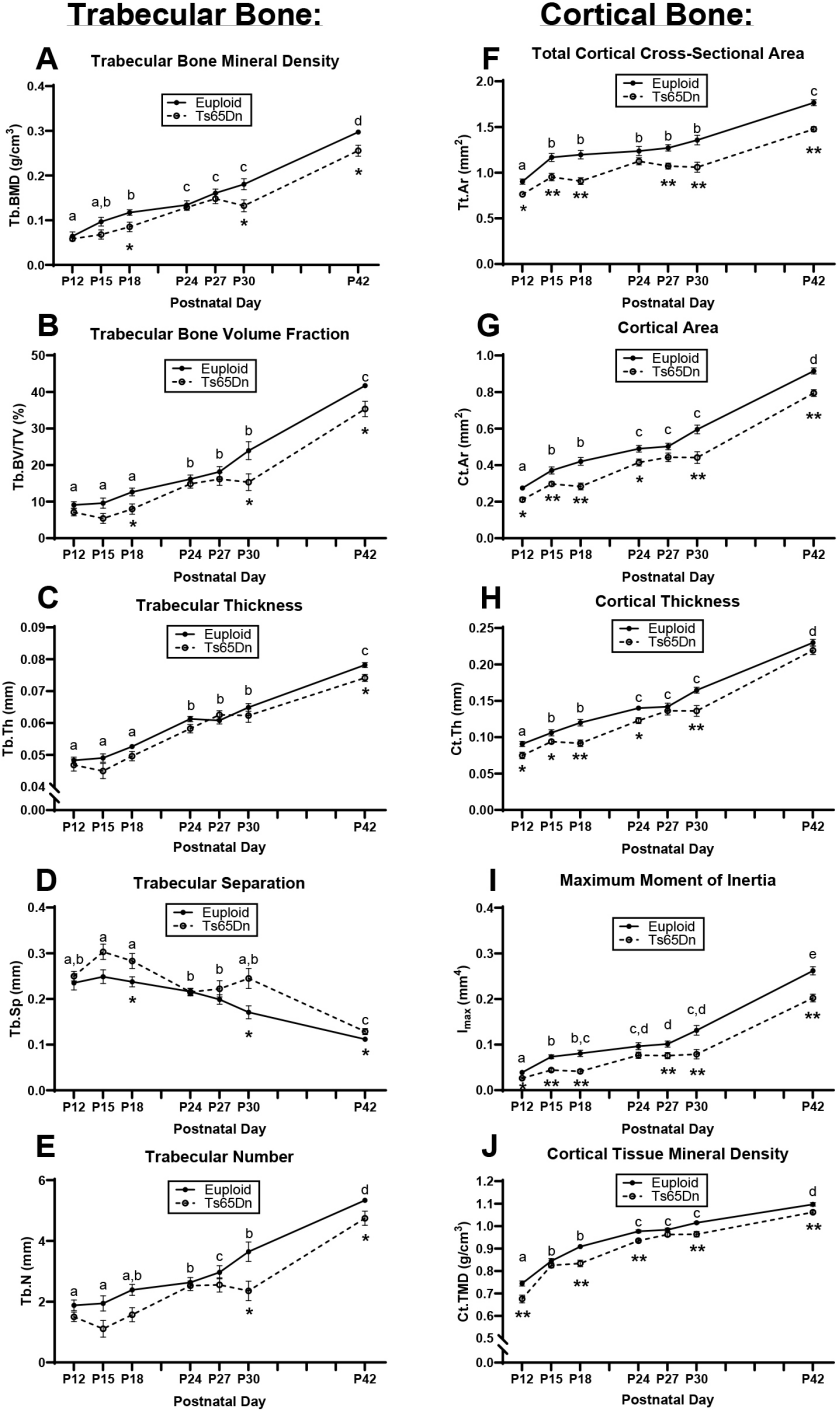
**Cross-sectional growth trajectory of trabecular and cortical bone in male euploid and Ts65Dn femurs from P12 to P42.** (A-J) Trabecular bone mineral density (Tb.BMD) (A), bone volume fraction (Tb.BV/TV) (B), thickness (Tb.Th) (C), separation (Tb.Sp) (D) and number (Tb.N) (E), and total cortical cross-sectional area (Tt.Ar) (F), cortical area (Ct.Ar) (G), cortical thickness (Ct.Th) (H), maximum moment of inertia (I_max_) and cortical tissue mineral density (Ct.TMD) (J) were assessed in male Ts65Dn mice and euploid littermate control mice from P12 to P42 (see Fig. S3 for other cortical bone parameters). Data are mean±s.e.m. See the legend of Fig. 1 for group numbers. Letters (a-e) indicate significant differences between ages by two-way ANOVA and Tukey’s or Games–Howell post hoc analysis (age effect); ages with the same letter are not significantly different from one another. * indicates adjusted *P*≤0.05 and ** indicates adjusted *P*≤0.01 by two-tailed unpaired *t*-test between euploid and Ts65Dn mice at the given age.

Trabecular and cortical data were then plotted on an age timeline from P12 to P42 and assessed by two-way ANOVA with age and genotype as factors to understand when trabecular and cortical bone growth were occurring. This perspective revealed stagnant periods of growth in euploid and Ts65Dn males with no significant growth (based on lack of age effect) from P12 to P18 and from P24 to P30 for most trabecular parameters (Fig. 3A-E). Stagnant periods of growth in cortical bone varied depending on the parameter in question: Tt.Ar, Ps.Pm and Ec.Pm did not significantly change from P15 to P30; Ma.Ar did not change from P15 to P42; and Ct.Ar, Ct.Th, I_max_, I_min_ and Ct.TMD had two stagnant periods from P15 to P18 and P24 to P30 (Fig. 3F-J; Fig. S3). Overall, this indicated two major periods of trabecular bone growth (P18 to P24, then P30 to P42) and three major periods of cortical bone growth (P12 to P15, then P18 to P24, then P30 to P42). These data prompted a deeper investigation into genetic mechanisms involved during the growth period spanning P12 to P42.

### Relationship between *Dyrk1a* expression and bone development in male Ts65Dn mice

**Fig. 4.**
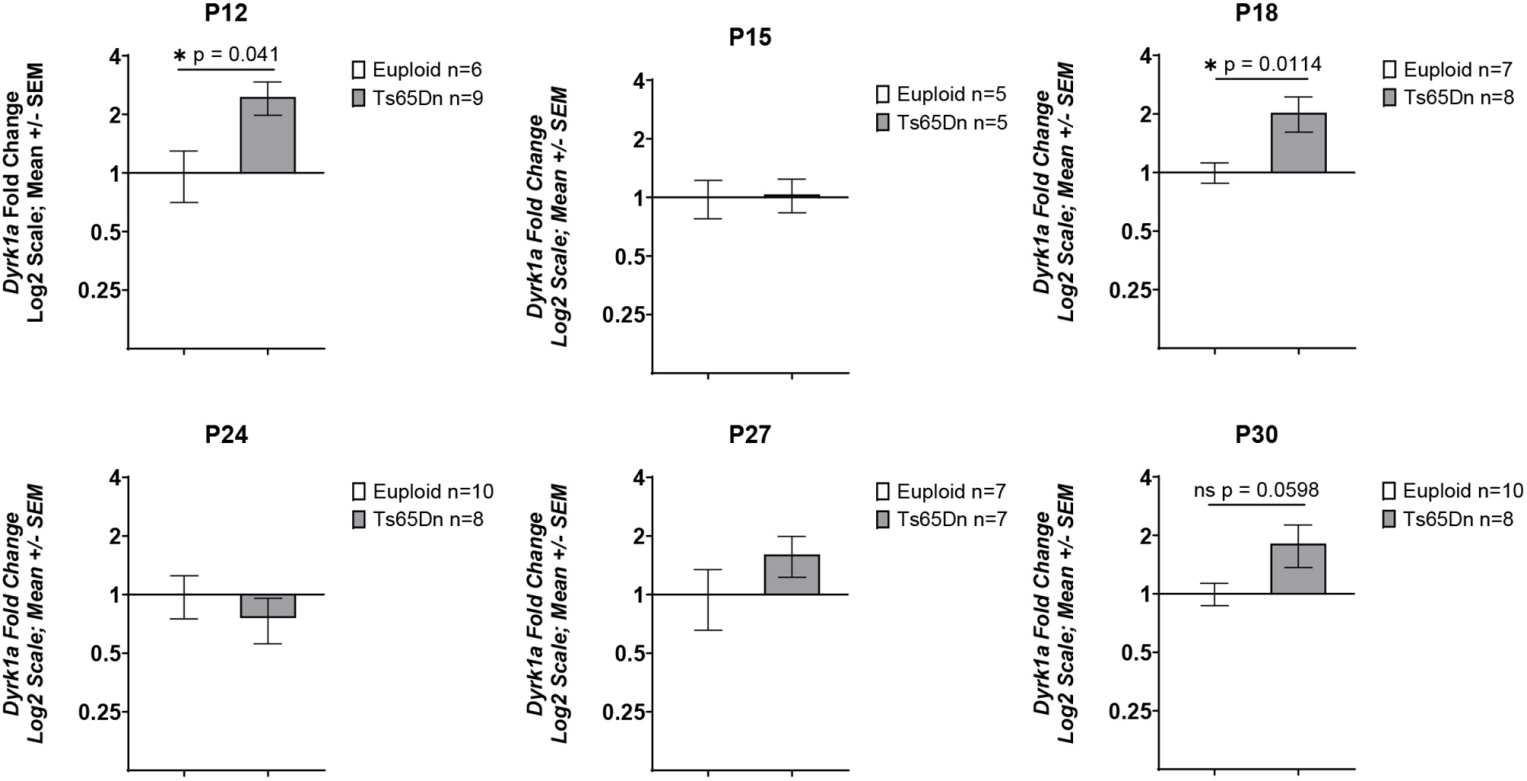
**Expression of *Dyrk1a* in Ts65Dn male mice from P12 to P42.** qPCR analyses of *Dyrk1a* cDNA from femoral midshafts obtained from P12, P15, P18, P24, P27 and P30 Ts65Dn and euploid male mice are represented by fold change on a log_2_ scale. Data are mean±s.e.m. P12, *n*=6 euploid and 9 Ts65Dn mice; P15, *n*=5 euploid and 5 Ts65Dn mice; P18, *n*=7 euploid and 8 Ts65Dn mice; P24, *n*=10 euploid and 8 Ts65Dn mice; P27, *n*=7 euploid and 7 Ts65Dn mice; P30, *n*=10 euploid and 8 Ts65Dn mice. *P<0.05 (one-tailed unpaired *t*-test).

We have previously shown that three copies of *Dyrk1a* is an important factor in causing trabecular and cortical cross-sectional area deficits in 6-week-old Ts65Dn male mice (Blazek et al., 2015a). To test the hypothesis that *Dyrk1a* overexpression during postnatal development in male Ts65Dn mice is linked to the emergence of appendicular skeletal abnormalities, RNA was isolated from the midshafts of left femurs obtained from P12, P15, P18, P24, P27 and P30 Ts65Dn and euploid male mice. At P12, male trisomic *Dyrk1a* was significantly overexpressed with a fold change of 2.053 (*P*=0.041) (Fig. 4). The male trisomic *Dyrk1a* fold change at P15 was 1.037 (*P*=0.417). *Dyrk1a* was significantly overexpressed at P18; the fold change for male Ts65Dn mice was 2.03 (*P*=0.011). At P24, the male trisomic *Dyrk1a* fold change was determined to be 0.76 (*P*=0.238). The *Dyrk1a* fold change at P27 for Ts65Dn male mice was 1.61 (*P*=0.072). The *Dyrk1a* fold change for Ts65Dn male mice at P30 was 1.81 (*P*=0.0598).

### Treatment of male Ts65Dn mice with CX-4945, a putative DYRK1A inhibitor

Given that the germline reduction of *Dyrk1a* in otherwise trisomic male mice resulted in improved trabecular phenotypes at P42 (Blazek et al., 2015a), significant femoral defects emerged at P30 after a period of stagnant development in male Ts65Dn mice, and *Dyrk1a* trended toward higher expression at P27 and P30, we hypothesized that treatment of male Ts65Dn animals with the DYRK1A inhibitor CX-4945 would improve skeletal formation at P30. Male euploid and Ts65Dn littermates were given 75 mg/kg/day CX-4945 suspension (90% PBS:10% DMSO) or vehicle (90% PBS:10% DMSO) via oral gavage from P21 to P29. Mice were euthanized and femurs were collected for micro-computed tomography (micro-CT) analysis on P30.

A repeated-measures ANOVA was performed on body weight data of vehicle- and CX-4945-treated euploid and Ts65Dn male mice, which showed an effect of age (*P*<0.001) and an interaction of age and treatment (*P*=0.014). Pairwise comparisons with a Bonferroni adjustment showed that the daily increments in body weight across the 10 days of treatment varied between the vehicle- and CX-4945-treated groups, but there were no significant weight differences between vehicle- and CX-4945-treated groups on any treatment day. Two-way ANOVA of average weight from P21 until P30 revealed a genotype effect where Ts65Dn male mice weighed less than euploid mice, which is typical for these mice at this age (Table S2). Weight gained during treatment (weight at P21 subtracted from weight at P30) showed no significant effects of genotype or treatment, nor their interaction, by two-way ANOVA.

Analysis of femoral structure revealed a main effect of genotype in trabecular BMD (*P*=0.008), BV/TV (*P*=0.008), Tb.N (*P*=0.008) and Tb.Sp (*P*=0.008), and in cortical Tt.Ar (*P*=0.005), Ma.Ar (*P*=0.005), Ct.Ar (*P*=0.005), Ct.Th (*P*=0.005), Ps.Pm (*P*=0.005), Ec.Pm (*P*=0.005), I_max_ (*P*=0.005), I_min_ (*P*=0.005) and Ct.TMD (*P*=0.005) (Table S2). These genotypic differences largely replicated what was previously observed at P30 for Ts65Dn male mice (Table S3). From these measures, treatment with CX-4945 from P21 to P29 did not improve skeletal defects at P30 in male Ts65Dn mice.

### Temporal reduction of *Dyrk1a* copy number in male Ts65Dn mice

Given the growth patterns from P24 to P30, the altered expression of trisomic *Dyrk1a* in developing mice, and the findings that normalization of *Dyrk1a* from conception to P42 in Ts65Dn animals restored trabecular and cortical skeletal phenotypes and that CX-4945 treatment did not correct skeletal deficits in male Ts65Dn mice, we hypothesized that the genetic normalization of *Dyrk1a* copy number at P21 would normalize skeletal development of Ts65Dn male mice at P30. To test this hypothesis, Ts65Dn mice were crossed with *Dyrk1a*^fl/wt^ mice and the progeny crossed with these doxycycline-inducible Cre promotor model rtTA^+^,tetOCre^+^. In the progeny, doxycycline administration activates Cre expression to excise exon 5 and 6 in one allele of *Dyrk1a*, leaving the expression product non-functional (Thompson et al., 2015). Doxycycline administration at weaning (P21) reduced *Dyrk1a* functional copy number in trisomic male Ts65Dn,*Dyrk1a*^fl/wt^, rtTA^+^,tetOCre^+^ (referred to as Ts65Dn,*Dyrk1a*^+/+/dox-cre^) mice during this time (Fig. S1).

At P30, there was an effect of genotype in trabecular BMD (*P*=0.003), BV/TV (*P*=0.003), Tb.N (*P*=0.003) and Tb.Sp (*P*=0.003), and in cortical Tt.Ar (*P*=0.002), Ma.Ar (*P*=0.002), Ct.Ar (*P*=0.002), Ct.Th (*P*=0.002), Ps.Pm (*P*=0.002), Ec.Pm (*P*=0.002), I_max_ (*P*=0.002), I_min_ (*P*=0.002) and Ct.TMD (*P*=0.002) (Table S4). There was no significant difference in *Dyrk1a* RNA expression among euploid, Ts65Dn and Ts65Dn,*Dyrk1a*^+/+/dox-cre^ mice (Fig. S4). The genotypic skeletal differences largely replicated those previously observed between Ts65Dn and euploid littermate male mice at P30 (Table S5), and normalization of *Dyrk1a* copy number, in otherwise trisomic Ts65Dn mice at P21, did not improve skeletal deficits at P30 in male Ts65Dn mice.

### Skeletal analysis at P30 of male and female mice with reduction of *Dyrk1a* copy number from conception

**Fig. 5.**
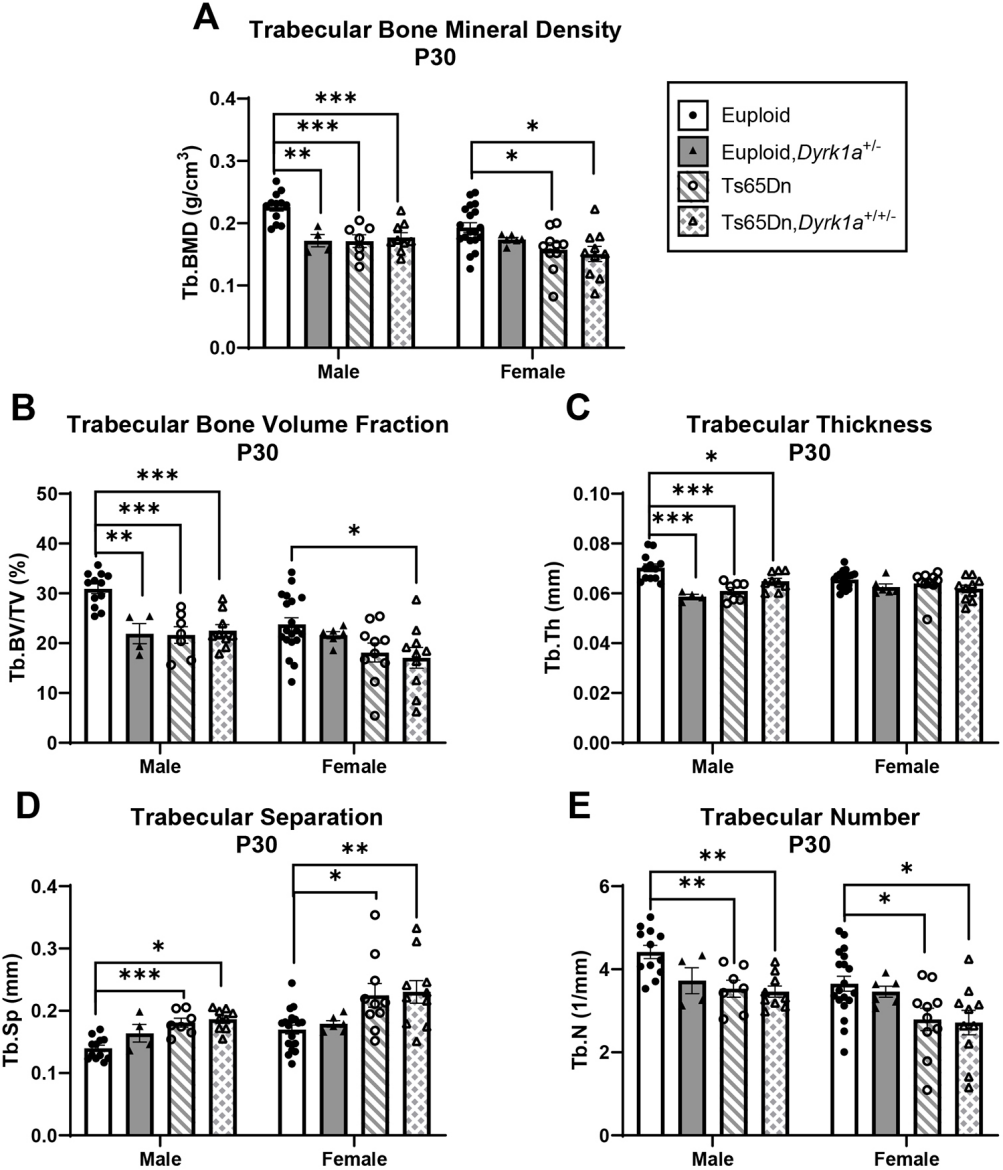
**Trabecular analysis of *Dyrk1a* normalization from conception in male and female euploid and Ts65Dn femurs at P30.** (A-E) Trabecular bone mineral density (Tb.BMD) (A), bone volume fraction (Tb.BV/TV) (B), thickness (Tb.Th) (C), separation (Tb.Sp) (D) and number (Tb.N) (E) were assessed in male and female euploid, euploid,*Dyrk1a^+/−^*, Ts65Dn and Ts65Dn,*Dyrk1a^+/−^* femurs at P30. Data are mean±s.e.m. Male: *n*=12 euploid, 4 euploid,*Dyrk1a*^+/−^, 7 Ts65Dn and 9 Ts65Dn,*Dyrk1a*^+/+/−^ mice. Female: *n*=19 euploid, 6 euploid,*Dyrk1a*^+/−^, 10 Ts65Dn and 10 Ts65Dn,*Dyrk1a*^+/+/−^ mice. Male and female mice were analyzed separately using one-way ANOVA and Tukey’s or Games–Howell post hoc analysis. *P≤0.05; **P≤0.01; ***P≤0.001.

**Fig. 6.**
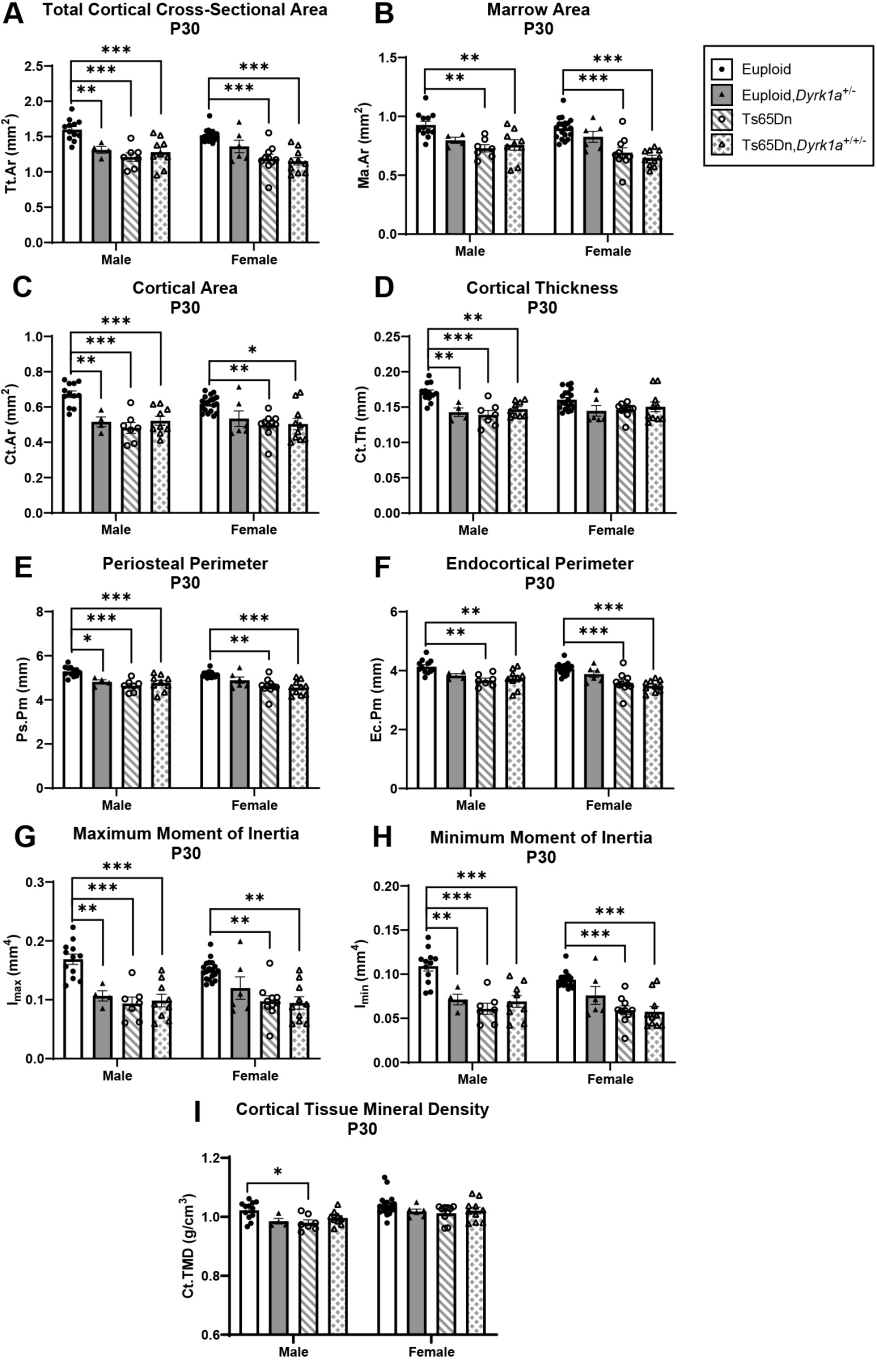
**Cortical analysis of *Dyrk1a* normalization from conception in male and female euploid and Ts65Dn femurs at P30.** (A-I) Total cortical cross-sectional area (Tt.Ar) (A), marrow area (Ma.Ar) (B), cortical area (Ct.Ar) (C), cortical thickness (Ct.Th) (D), periosteal perimeter (Ps.Pm) (E), endocortical perimeter (Ec.Pm) (F), maximum moment of inertia (I_max_) (G), minimum moment of inertia (I_min_) (H) and cortical tissue mineral density (Ct.TMD) (I) were assessed in male and female euploid, euploid,*Dyrk1a^+/−^*, Ts65Dn and Ts65Dn,*Dyrk1a^+/−^* femurs at P30. Data are mean±s.e.m. Male: *n*=12 euploid, 4 euploid,*Dyrk1a*^+/−^, 7 Ts65Dn and 9 Ts65Dn,*Dyrk1a*^+/+/−^ mice. Female: *n*=19 euploid, 6 euploid,*Dyrk1a*^+/−^, 10 Ts65Dn and 10 Ts65Dn,*Dyrk1a*^+/+/−^ mice. Male and female mice were analyzed separately using one-way ANOVA and Tukey’s or Games–Howell post hoc analysis. *P≤0.05; **P≤0.01; ***P≤0.001.

Due to previous observations of significant improvement of trabecular phenotypes in conceptionally reduced Ts65Dn,*Dyrk1a*^+/+/−^ mice (Blazek et al., 2015a) and our current observations of both *Dyrk1a* expression and skeletal development, we hypothesized that a reduction of *Dyrk1a* from conception in otherwise trisomic mice would lead to correction of skeletal deficits in Ts65Dn male mice at P30. Additionally, we wanted to test the hypothesis that female Ts65Dn mice did not have affected trabecular or cortical measures at P30, because female humans with DS exhibit bone deficits later than males (Carfi et al., 2017). To test these hypotheses, femoral skeletal properties were quantified in offspring from Ts65Dn×*Dyrk1a^+/−^* matings at P30. There was a significant genotype effect in all trabecular and cortical measures in male mice from a one-way ANOVA: BMD, BV/TV, Tb.Th, Tb.N and Tb.Sp (*P*=0.002 for all measures), and Tt.Ar, Ma.Ar, Ct.Ar, Ct.Th, Ps.Pm, Ec.Pm, I_max_, I_min_ and Ct.TMD (*P*=0.002 for all measures) (Figs 5 and 6). At P30, Tukey's post hoc analyses found that euploid skeletal measures were greater than those of Ts65Dn, Ts65Dn,*Dyrk1a*^+/+/−^ and euploid,*Dyrk1a*^+/−^ male mice for all trabecular and cortical measures except for Tb.Sp, which was less in euploid than in Ts65Dn and Ts65Dn,*Dyrk1a*^+/+/−^ mice, and cortical Ma.Ar and Ec.Pm, which were greater in euploid than in Ts65Dn and Ts65Dn,*Dyrk1a*^+/+/−^ mice. Tukey's post hoc analyses from one-way ANOVA comparison of just Ts65Dn, Ts65Dn,*Dyrk1a*^+/+/−^ and euploid mice showed that euploid mice were significantly different from both trisomic genotypes.

For female mice, comparing all offspring from Ts65Dn×*Dyrk1a^+/−^* matings at P30 found significant genotype effects in BMD (*P*=0.018), BV/TV (*P*=0.020), Tb.N (*P*=0.015) and Tb.Sp (*P*=0.005), and in Tt.Ar, Ma.Ar, Ct.Ar, Ps.Pm, Ec.Pm, I_max_ and I_min_ (*P*=0.002 for all measures) (Figs 5 and 6). Tukey's post hoc analyses found that BMD and Tb.N were greater in euploid mice than in both Ts65Dn and Ts65Dn,*Dyrk1a*^+/+/−^ mice (Fig. 5A,E). BV/TV was significantly greater in euploid mice than in Ts65Dn,*Dyrk1a*^+/+/−^ mice (Fig. 5B). Tb.Sp was reduced in euploid mice compared to that in Ts65Dn and Ts65Dn,*Dyrk1a*^+/+/−^ mice (Fig. 5D). Tukey's or Games-Howell's post hoc analyses for cortical measures found that these were greater in euploid female mice than in both Ts65Dn and Ts65Dn,*Dyrk1a*^+/+/−^ mice (Fig. 6). Taking all data together, Ts65Dn male and female mice exhibited significant deficits in trabecular and cortical bone at P30; these deficits persisted even when *Dyrk1a* copy number was returned to normal levels from conception in otherwise trisomic Ts65Dn mice.

### Skeletal analysis at P36 of male and female mice with germline reduction of *Dyrk1a* copy number

**Fig. 7.**
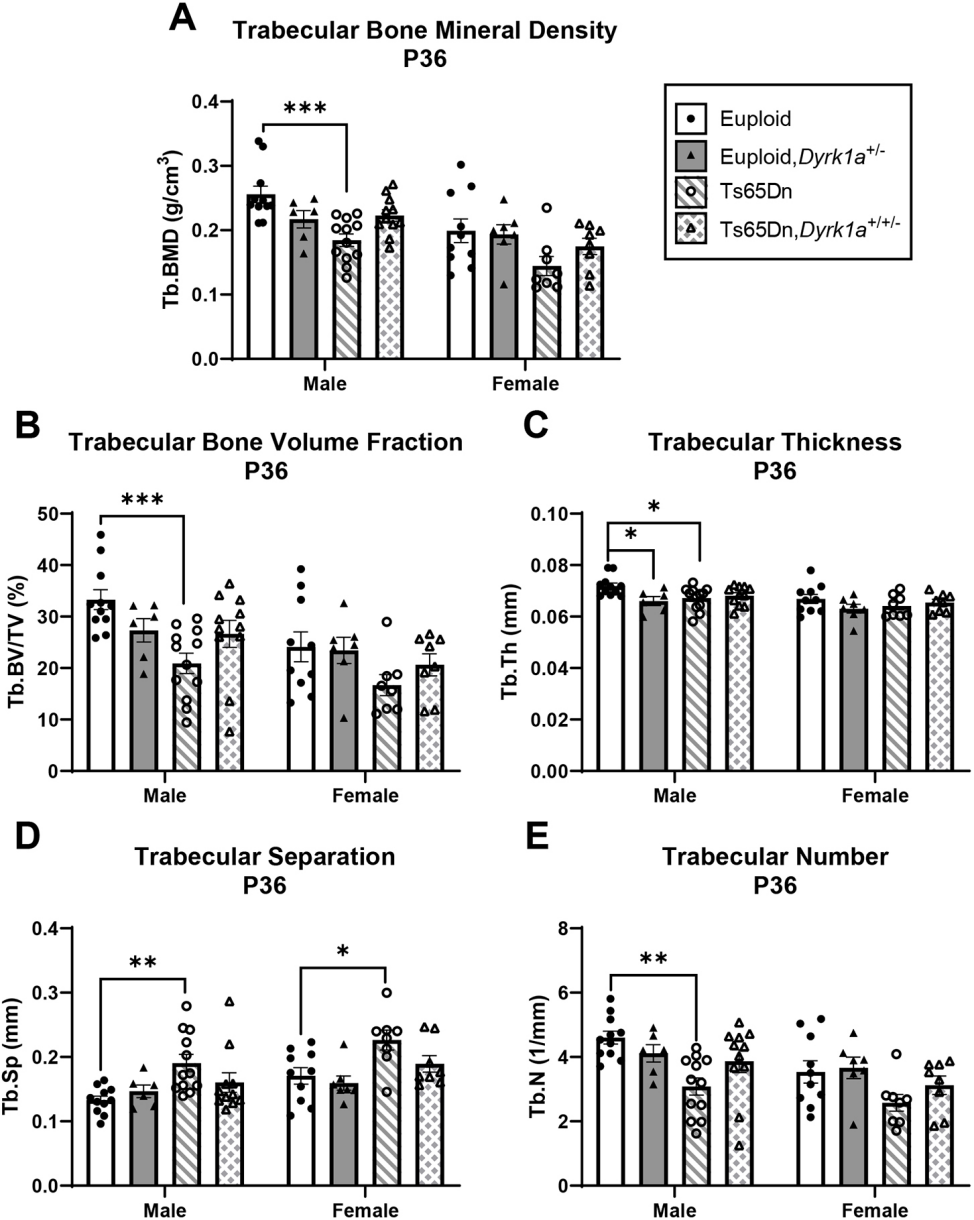
**Trabecular analysis of *Dyrk1a* normalization from conception in male and female euploid and Ts65Dn femurs at P36.** (A-E) Trabecular bone mineral density (Tb.BMD) (A), bone volume fraction (Tb.BV/TV) (B), thickness (Tb.Th) (C), separation (Tb.Sp) (D) and number (Tb.N) (E) were assessed in male and female euploid, euploid,*Dyrk1a^+/−^*, Ts65Dn and Ts65Dn,*Dyrk1a^+/−^* femurs at P36. Data are mean±s.e.m. Male: *n*=11 euploid, 6 euploid,*Dyrk1a*^+/−^, 12 Ts65Dn and 11 Ts65Dn,*Dyrk1a*^+/+/−^ mice. Female: *n*=10 euploid, 7 euploid,*Dyrk1a*^+/−^, 8 Ts65Dn and 8 Ts65Dn,*Dyrk1a*^+/+/−^ mice. Male and female mice were analyzed separately using one-way ANOVA and Tukey’s or Games–Howell post hoc analysis. *P≤0.05; **P≤0.01; ***P≤0.001.

**Fig. 8.**
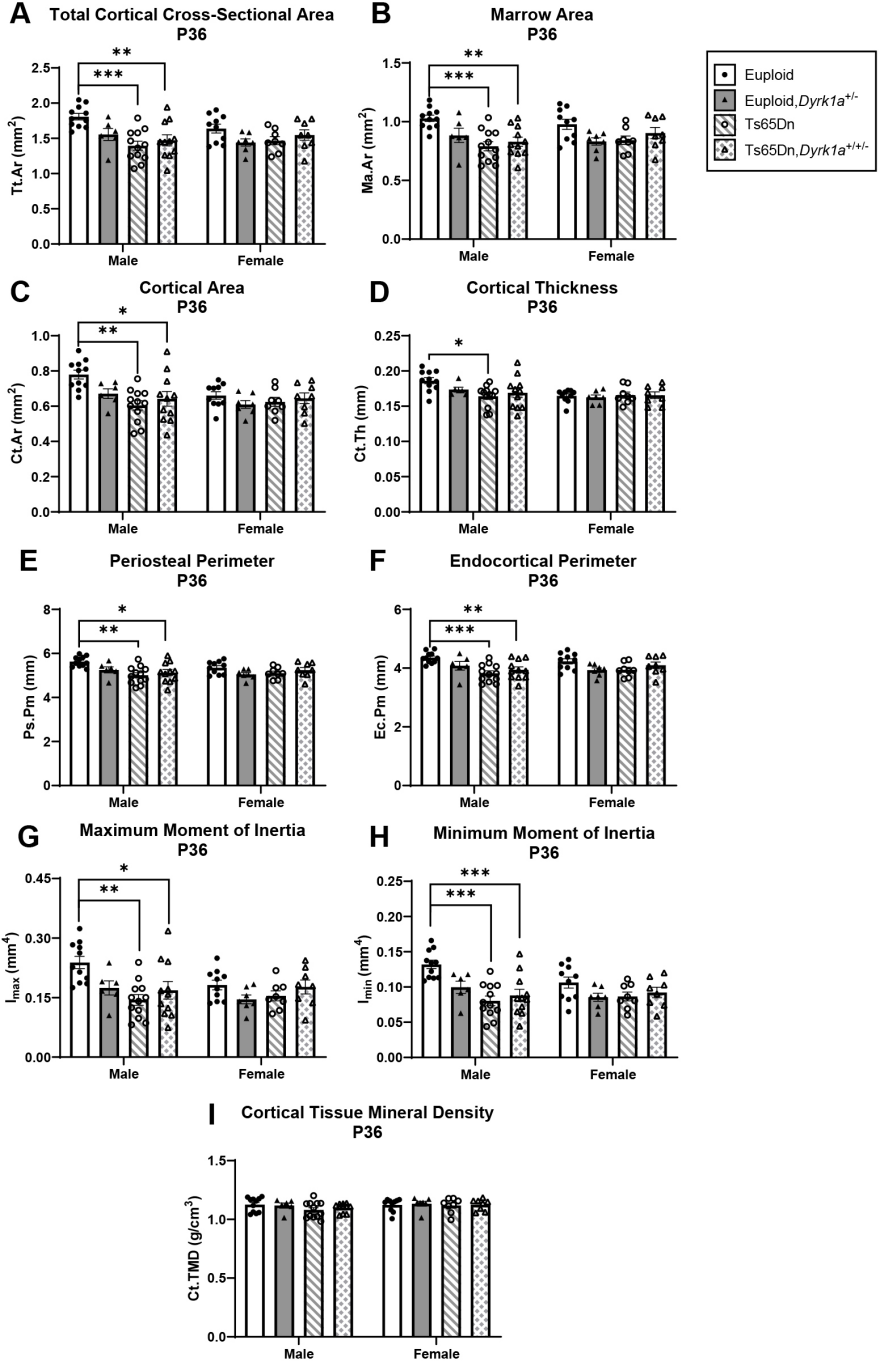
**Cortical analysis of *Dyrk1a* normalization from conception in male and female euploid and Ts65Dn femurs at P36.** (A-I) Total cortical cross-sectional area (Tt.Ar) (A), marrow area (Ma.Ar) (B), cortical area (Ct.Ar) (C), cortical thickness (Ct.Th) (D), periosteal perimeter (Ps.Pm) (E), endocortical perimeter (Ec.Pm) (F), maximum moment of inertia (I_max_) (G), minimum moment of inertia (I_min_) (H) and cortical tissue mineral density (Ct.TMD) (I) were assessed in male and female euploid, euploid,*Dyrk1a^+/−^*, Ts65Dn and Ts65Dn,*Dyrk1a^+/−^* femurs at P36. Data are mean±s.e.m. Male: *n*=11 euploid, 6 euploid,*Dyrk1a*^+/−^, 12 Ts65Dn and 11 Ts65Dn,*Dyrk1a*^+/+/−^ mice. Female: *n*=10 euploid, 7 euploid,*Dyrk1a*^+/−^, 8 Ts65Dn and 8 Ts65Dn,*Dyrk1a*^+/+/−^ mice. Male and female mice were analyzed separately using one-way ANOVA and Tukey’s or Games–Howell post hoc analysis. *P≤0.05; **P≤0.01; ***P≤0.001.

To understand whether the skeletal deficits persisted in both male and female Ts65Dn mice and whether normalization of *Dyrk1a* copy number would begin to correct these deficits at P36 (halfway between P30 when no correction was seen and P42 when a previous correction was observed), trabecular and cortical skeletal phenotypes were quantified in the offspring of Ts65Dn×*Dyrk1a^+/−^* matings at P36. For male mice, we found both trabecular [BMD (*P*=0.005), BV/TV (*P*=0.006), Tb.Th (*P*=0.021), Tb.N (*P*=0.006) and Tb.Sp (*P*=0.013)] and cortical [Tt.Ar (*P*=0.004), Ma.Ar (*P*=0.004),Ct.Ar (*P*=0.003), Ct.Th (*P*=0.026), Ps.Pm (*P*=0.003), Ec.Pm (*P*=0.004), I_max_ (*P*=0.003) and I_min_ (*P*=0.004)] measures were significantly different by one-way ANOVA (Figs 7 and 8). In a post hoc analysis using Tukey's test, euploid male mice were only different from Ts65Dn male mice for BMD, Tb.Th, Tb.N and Tb.Sp (and euploid,*Dyrk1a*^+/−^ mice for Tb.Th), indicating potential improvement in trabecular bone when *Dyrk1a* copy number was returned to normal in male Ts65Dn mice at P36 (Fig. 7). For cortical deficits, all measures were greater in euploid mice that those in both Ts65Dn and Ts65Dn,*Dyrk1a*^+/+/−^ mice except for Ct.Th, which was only significantly greater in euploid mice than in Ts65Dn mice, indicating largely no effect of *Dyrk1a* normalization in cortical bone of male Ts65Dn mice at P36 (Fig. 8).

For female offspring of Ts65Dn×*Dyrk1a^+/−^* matings at P36, the only difference observed was Tb.Sp; euploid and euploid,*Dyrk1a*^+/−^ mice had significantly less Tb.Sp than Ts65Dn and Ts65Dn,*Dyrk1a*^+/+/−^ mice (Fig. 7D). No other trabecular or cortical parameters were significantly different in female offspring of Ts65Dn×*Dyrk1a^+/−^* matings at P36 (Figs 7 and 8).

To better understand how other influences besides *Dyrk1a* copy number affected trisomic bone development from P24 to P30, during the stagnant growth that results in lasting defects, we examined mRNA expression in femurs from genes that have been linked to skeletal development, i.e. *Bglap*, *Runx2*, *Rbl2* and *Alpl*, at P24, P27 and P30 (Rodda and McMahon, 2006; Gao et al., 2014). *Rbl2*, also known as *P130*, is a gene transcribed via the transcription factor FOXO1 (Kops et al., 2002). Nuclear FOXO1 competes with TCF/LEF1 for β-catenin to transcribe target genes but is also exported from the nucleus after phosphorylation by DYRK1A (Bhansali et al., 2021). *Rbl2* is hypothesized to indirectly report DYRK1A activity (Forristal et al., 2014); increased DYRK1A activity would result in reduced expression of *Rbl2*. In trisomic male Ts65Dn mice at P24, P27 and P30, *Bglap*, *Runx2*, *Rbl2* and *Alpl* levels were not statistically different than those in euploid littermate mice (Fig. S5).

## DISCUSSION

### Initiation of bone deficits in male Ts65Dn mice

Male individuals with DS have bone deficits at earlier ages than female individuals with DS compared to those without DS. Skeletal deficits in Ts65Dn mice have shown a similar trend, with persistent skeletal deficits occurring in male trisomic mice earlier than in female mice. This study of male Ts65Dn mice during postnatal development shows that cortical deficits are present in male trisomic mice compared to euploid mice beginning at P12, and some trabecular deficits begin to present at P18. Some trisomic trabecular and cortical measurements are nearly equivalent to those in euploid male mice at P24 and appear to exhibit stagnant development in male Ts65Dn mice until P30, when male trisomic trabecular and cortical deficits are fully present again. In male Ts65Dn mice, trabecular and cortical deficits persist at P36, and as we have previously shown (Blazek et al., 2011), at P42 (6 weeks) and 16 weeks. Others have noted similar trabecular and cortical deficits in tibiae of male Ts65Dn mice at 8 weeks and 24 months (Fowler et al., 2012; Williams et al., 2018). Taken together, in male Ts65Dn mice, cortical bone deficits were present at almost every developmental age tested, whereas trabecular deficits were more transient, but increased significantly during development. The oscillations in trabecular phenotypes in male mice might be due to altered expression of *Dyrk1a* and/or other trisomic genes during certain timepoints based on our quantitative PCR (qPCR) analysis of bone. It is unknown if a similar pattern of oscillating bone phenotypes is seen in humans with DS because longitudinal long bone studies of trabecular bone have not been completed in humans with DS. This could partially explain inconsistencies between studies of bone phenotypes in adolescents with DS.

### Differences in skeletal deficits between trisomic male and female mice

In previous findings, we demonstrated that at P42, female Ts65Dn mice showed inferior femoral trabecular properties in BMD and Tb.Sp, and also showed reduced cortical measures including Tt.Ar, Ma.Ar, Ps.Pm and Ec.Pm, compared to female euploid mice (Thomas et al., 2021). At P30, we observed that female trisomic mice from a Ts65Dn×*Dyrk1a*^+/−^ cross had deficits in femoral trabecular BMD, Tb.N and Tb.Sp and most cortical skeletal measures compared to euploid mice. At P36, however, femurs from female trisomic mice only had increased Tb.Sp and no significant cortical deficits. These data indicate that female trisomic mice have some appendicular bone deficits at P30, followed by a period of near-normal bone characteristics at P36, which become significantly worse by P42. Taken together, both trisomic male and female mice have periods of significant femoral deficits, periods of recovery to normal measures in a sex-specific manner (around P24 for males and P30 for females), which then become worse over time, with male Ts65Dn mice showing consistent bone deficits earlier (around P30) than female mice (between P36 and P42).

In Dp1Tyb mice, a duplication mouse model of DS, trabecular deficits also are observed in male Dp1Tyb mice at P42 before female Dp1Tyb mice (Thomas et al., 2020), consistent with Ts65Dn mice. However, cortical deficits are present in both sexes at all ages investigated in Dp1Tyb mice, which is inconsistent with Ts65Dn mice, but this may be due to the limited number of timepoints investigated in both sexes of Dp1Tyb mice. Female Dp1Tyb mice are also discordant from female Ts65Dn mice in that they have not been found to have trabecular deficits, even at 16 weeks.

### Relationship of trisomic *Dyrk1a* to bone deficits in Ts65Dn mice

Three copies of *Dyrk1a* have been hypothesized to contribute to bone deficits and these data further clarify the timing of trisomic *Dyrk1a* involvement in appendicular skeletal deficits in Ts65Dn mice. Trisomic *Dyrk1a* was not consistently overexpressed in the femoral RNA of male mice at all timepoints from P12 to P30. We have shown that, in brains of Ts65Dn mice, DYRK1A protein and *Dyrk1a* mRNA are not always consistently overexpressed in the cerebellum, hippocampus and cerebral cortex throughout development (Hawley et al., 2023). Although *Dyrk1a* RNA is significantly overexpressed in the femurs of male Ts65Dn mice compared to its expression in control mice at P12 and P18 and trends to higher levels at P27 and P30, the normalization of *Dyrk1a* copy number in otherwise trisomic male mice from P21 to P30, or from conception, did not have a corrective effect on male or female trisomic trabecular or cortical femoral phenotypes at P30. At P36, there was no effect of trisomy or normalization of *Dyrk1a* copy number from conception in otherwise trisomic female mice, but in Ts65Dn,*Dyrk1a*^+/+/−^ male mice, trabecular deficits and Ct.Th were improved. Previous data from our laboratory showed that not all appendicular skeletal measures, cortical measures in particular, were affected by normalizing *Dyrk1a* expression (Blazek et al., 2015a; Sloan et al., 2023). Similarly, there is no apparent connection between oscillating *Dyrk1a* expression and cortical femoral phenotypes in perinatal development. Taken together, these data indicate that: (1) trisomic *Dyrk1a* expression changes throughout perinatal development in the femur, challenging the dogma in the field that all genes are consistently upregulated by 1.5-fold in trisomic mice at all times; (2) trisomic *Dyrk1a* begins to have a significant effect on trabecular and some cortical femoral phenotypes in Ts65Dn male mice at P36, even though there are ages at which *Dyrk1a* is overexpressed or trends toward overexpression in this bone; and (3) the femoral bone is likely affected in female Ts65Dn mice after P36 but before P42, yet the effect of trisomic *Dyrk1a* on female trisomic femurs is still unknown. How trisomic *Dyrk1a* affects skeletal phenotypes may change as Ts65Dn mice age, and the interaction among other trisomic genes may affect the developing trabecular and cortical deficits in at least male Ts65Dn mice (Sloan et al., 2023).

### CX-4945 treatment in male Ts65Dn mice

Using CX-4945, we tested the hypothesis that treatment using a putative DYRK1A inhibitor during a window of *Dyrk1a* overexpression and stagnating trisomic femoral development would improve the femoral bone in Ts65Dn mice. Crystal structures show that CX-4945 occupies the ATP-binding pocket of the kinase domain of DYRK1A, and *in vitro* assays of CX-4945 reduced cyclin D1 phosphorylation and NFAT signaling activity (Grygier et al., 2023). CX-4945 was not soluble in water or PBS, limiting initial dissolution to DMSO. Due to health concerns over multiple treatments with DMSO, the total percentage of each treatment in this study was limited to 10% total DMSO. CX-4945 did not remain dissolved at 90% PBS:10% DMSO; however, it formed a suspension that was relatively stable at 37°C. Preliminary intraperitoneal and subcutaneous injections of CX-4945 from P21 to P29 resulted in yellow precipitate at the injection site, suggesting that the drug precipitated at the injection site and was not bioavailable. Oral gavage trials were more convincing; organs seemed unaffected, feces were not discolored, and the gastric system appeared normal. Treatment of male Ts65Dn mice from P21 to P29 with 75 mg/kg/day CX-4945 in a 90% PBS:10% DMSO suspension via oral gavage, by which CX-4945 did not seem to adversely affect the organs, proved ineffective in improving *Dyrk1a*-related femoral phenotypes. This lack of improvement in the femur could be because of the limited effect of CX-4945 as a DYRK1A inhibitor in bone or because treatment was given when overexpression of *Dyrk1a* did not appear to affect bone development.

More unexpected was the effect of vehicle DMSO treatment on Ts65Dn mice; the mean BV/TV in vehicle-treated mice was approximately 23%, compared to 15% in untreated P30 Ts65Dn mice characterized earlier (Table S6). Vehicle treatment of male Ts65Dn mice from P21 to P29 appeared to improve trabecular measures to euploid levels. However, not all *Dyrk1a*-related femoral phenotypes were affected; Ct.Th was not significantly different between vehicle-treated and untreated Ts65Dn mice, suggesting a positive effect only on trabecular bone. Previous reports administering DYRK1A inhibitors via oral gavage showed significant differences in *Dyrk1a*-related measures in Ts65Dn mice receiving the vehicle (Goodlett et al., 2020; Stringer et al., 2017a), suggesting that 10% DMSO, as part of the vehicle treatment, improved trabecular defects.

DMSO has been reported to decrease osteoclast maturation in a dose-dependent manner (Lemieux et al., 2011; Yang et al., 2015). Others have reported increased osteoblast differentiation and activity in mouse and human osteoblasts treated with DMSO, along with increased osteogenic gene expression (Stephens et al., 2011; Cheung et al., 2006). It may be that DMSO interacts with products of trisomy to significantly increase femoral measures in Ts65Dn but not euploid mice. Although unlikely, it is possible that because the CX-4945-treated cohorts were treated before vehicle-treated cohorts, seasonal growth effects impacted analysis between the two groups. It is possible that nutritional aspects play a role; mean body weight was increased at P30 in vehicle-treated Ts65Dn mice (15.72 g) compared to that in untreated Ts65Dn mice (12.74 g), but there was no difference between vehicle-treated (18.58 g) and untreated (19.63 g) euploid mice (Table S6). This illustrates the importance of using vehicle treatment in therapeutic studies on DS and other models.

### Implications for treatments of bone deficits

Overexpression of *Dyrk1a* during crucial developmental stages likely contributes to DS-related phenotypes such as cognitive impairment, skeletal deficits and craniofacial abnormalities (Hawley et al., 2023; Stringer et al., 2017b; McElyea et al., 2016). Although interventions targeting *Dyrk1a* overexpression using genetic and therapeutic mechanisms have shown limited normalization of some skeletal and neurodevelopmental deficits, the potential efficacy of translational DYRK1A inhibitors is still questionable (Stringer et al., 2017b). We have shown that expression of trisomic *Dyrk1a* varies spatially and temporally in the bone and brain and may affect a wide range of DS-associated phenotypes. Because of the dynamic nature of *Dyrk1a* overexpression and the dosage effects of both over- and under-expression of *Dyrk1a*, care must be taken when administering DYRK1A inhibitors. Variants in *DYRK1A* may lead to DYRK1A syndrome that includes both neurological and skeletal phenotypes, including short stature, microcephaly and tibial osteochondrosis (Ji et al., 2015; Huang et al., 2023; Meissner et al., 2020). Additionally, we have shown trabecular and cortical bone deficits in P30 and P42 male mice with only one functional copy of *Dyrk1a* from conception (Figs 5 and 6) (Blazek et al., 2015a). A general pharmacological reduction of DYRK1A, especially during development, may have detrimental effects in tissues in which *Dyrk1a* is not overexpressed. Administration of a DYRK1A inhibitor during an incorrect window of time may not improve the targeted deficit, whereas a later administration of the inhibitor may have positive effects. A negative result may not be due to the limited efficacy or off-target effects of a potential DYRK1A inhibitor, but rather to incorrect timing and tissue specificity of the inhibitor.

Additionally, differences in expression of trisomic *Dyrk1a* between the sexes must be accounted for when determining therapeutic approaches. Differential expression of DYRK1A protein and *Dyrk1a* RNA between male and female Ts65Dn mice has been shown in the cerebellum, cerebral cortex and hippocampus (Hawley et al., 2022, 2023), and the differential effects of trisomic *Dyrk1a* on femur phenotypes is now apparent (this study). From these studies of trisomic *Dyrk1a* expression, it appears that treatments to inhibit DYRK1A would need to differ between the sexes because of the spatiotemporal differences in *Dyrk1a* overexpression. Additionally, attempted normalization of DYRK1A expression would need to begin in males (before P36) before females and continue through the major influences of trisomic DYRK1A on bone formation to correct femoral deficits.

### Study limitations

There are many DS mouse models in which bone deficits have been characterized, including Ts65Dn, Dp1Tyb, Dp1Rhr and Dp(16)1Yey (Blazek et al., 2011; Thomas et al., 2020; Sherman et al., 2022; Sloan et al., 2023). Ts65Dn mice are the most characterized DS mouse model and appear to effectively model femoral DS-associated skeletal deficits, but skeletal studies in female mice have been limited because of the need to use these mice to generate additional mice. Furthermore, Ts65Dn mice also contain triplication of ∼35 protein-coding genes that are not orthologous to those in Hsa21. The recently generated Ts66Yah mouse removes these non-orthologous genes (Duchon et al., 2022), and skeletal abnormalities in these mice have not yet been characterized. Additionally, offspring used to identify the influence of trisomic *Dyrk1a* in the study of *Dyrk1a* skeletal deficits from P12 to P30 came from Ts65Dn×B6C3.*Dyrk1a^fl/fl^* matings. Ts65Dn and euploid mice should be equivalent on the ∼50% B6 and ∼50% C3H background from these studies, but there may have been intrauterine influences from male mice used in these crosses.

Attempts to normalize the skeleton of Ts65Dn mice between P21 and P30 by a temporal reduction of *Dyrk1a* were not successful. Besides our interpretation of this time representing an earlier than necessary window of normalizing *Dyrk1a*, other technical or biological issues may have also contributed to these approaches not achieving the desired correction, including an incomplete deletion of the third copy of *Dyrk1a*, a long half-life of *Dyrk1a* RNA or DYRK1A protein, or not having sufficient replicates to detect small corrections. These issues may have also contributed to the inability to normalize *Dyrk1a* RNA after the temporal reduction. Further analyses may resolve these issues.

Owing to the composition of the bones at these ages, functional analysis (three-point mechanical bending) could not be performed; thus, the direct strength of these bones was not measured. However, I_max_ and I_min_, calculated from cortical cross-sections, can be used as an indication of the response of the bone to force and to estimate bone strength (Cole and van der Meulen, 2011; Wallace, 2019).

### Conclusion

This study identifies key timepoints in appendicular skeletal development in the Ts65Dn DS mouse model for targeted treatment of skeletal deficits associated with triplicated *Dyrk1a*. Male Ts65Dn mice have cortical deficits earlier (P12) than trabecular deficits, which are not consistently altered until P30. Periodic normalization of femoral phenotypes was also identified in male and female Ts65Dn mice. Despite having triplicated *Dyrk1a*, *Dyrk1a* mRNA levels are not always overexpressed in male Ts65Dn femurs. *Dyrk1a* normalization improves skeletal phenotypes in male mice after P30, and this effect is largely limited to the trabecular bone. This indicates a complex relationship between *Dyrk1a* expression and skeletal phenotypes associated with Ts21 that may be influenced by several factors including age, sex and timing of *Dyrk1a* overexpression. Our data from female Ts65Dn mice suggest that consistent trabecular and cortical deficits may not arise until P42 (after they arise in male Ts65Dn mice), but later ages need to be analyzed to confirm this. Overall, this study illustrates the importance of identifying spatial and temporal windows of development and gene expression in DS mouse models to improve preclinical treatment outcomes.

## MATERIALS AND METHODS

### Animal models

Ts(17^16^)65Dn (Ts65Dn) female (stock 001924; ∼50% C57BL/6 and ∼50% C3H/HeJ advanced intercross background) and B6C3F1 male (stock 100010) mice (*Mus musculus*) were obtained from The Jackson Laboratory. New Ts65Dn females and B6C3F1 males from The Jackson Laboratory were added to the colony approximately every 6 months to reduce strain variability. *Dyrk1a* heterozygous mutant mice (*Dyrk1a*^+/−^) were obtained from Dr Mariona Arbones [Centro de Investigación Biomédica en Red de Enfermedades Raras (CIBERER) and Institut de Biologia Molecular de Barcelona (IBMB), Barcelona, Spain] (Fotaki et al., 2002, 2004) and were subsequently backcrossed to B6C3F1 mice for more than ten generations to parallel the genetic background of Ts65Dn mice. B6*.Dyrk1a^tm1Jdc^* (*Dyrk1a^fl/fl^*) mice containing *loxP* sites flanking *Dyrk1a* exons 5 and 6 were obtained from Dr John Crispino (St. Jude Children's Research Hospital, Memphis, TN) (Thompson et al., 2015) and bred with C3H/HeJ mice (The Jackson Laboratory, stock 000659), resulting in B6C3F1.*Dyrk1a^fl/wt^* offspring, containing *loxP* insertions on one *Dyrk1a* allele. These heterozygous offspring were intercrossed to produce homozygous B6C3.*Dyrk1a^fl/fl^* mice on a similar B6C3 advanced intercross genetic background as that of the Ts65Dn model. Unless otherwise noted, trisomic and euploid (control) offspring from Ts65Dn×B6C3.*Dyrk1a^fl/fl^* matings were used in these experiments.

B6N.FVB(Cg)-Tg(CAG-rtTA3)4288Slowe/J (The Jackson Laboratory, stock 016532) reverse tetracycline transactivator (rtTA) mice and B6.Cg-Tg(tetO-cre)1Jaw/J (The Jackson Laboratory, stock 006234) mice were first intercrossed with C3H/HeJ mice to produce founder strains on a 50% B6 and 50% C3H background similar to Ts65Dn mice. The B6C3F1 founder strains rtTa and tetO-cre were then crossed. Male progeny from this cross that were positive for both rtTA and tetO-cre were mated to Ts65Dn,*Dyrk1a*^fl/wt^ females to generate test mice that would experience the loss of a floxed *Dyrk1a* allele after treatment with doxycycline by inducing activation of the tetracycline-responsive promotor element and excision of exons 5 and 6 of *Dyrk1a* by Cre on one Mmu16.

Mice were group-housed with mixed genotypes according to sex in a 12 h:12 h light:dark cycle with white light off between 19:00 and 07:00. Femurs obtained from mice euthanized at specified timepoints were wrapped in gauze, soaked in phosphate-buffered saline (PBS), immersed quickly in liquid nitrogen, and stored at −80°C until ready to use. Right femurs were subjected to micro-CT analysis and left femurs were reserved for gene expression analysis. Experiments with animals were carried out in accordance with the National Institutes of Health Guide for the Care and Use of Laboratory Animals and received prior approval from the Institutional Animal Care and Use Committee at the Indiana University–Purdue University Indianapolis School of Science (SC298R and SC338R).

### Genotyping

Ts65Dn mice were genotyped to determine the presence of a freely segregating chromosome by amplifying the Mmu16/Mmu17 breakpoint using the primers 5ʹ-GTGGCAAGAGACTCAAATTCAAC-3ʹ and 5ʹ-TGGCTTATTATTATCAGGGCATTT-3ʹ (Reinholdt et al., 2011). *Dyrk1a*^fl/wt^ mice were genotyped by PCR as described by The Jackson Laboratory protocol using the primers 5ʹ-TACCTGGAGAAGAGGGCAAG-3ʹ and 5ʹ-GGCATAACTTGCATACAGTGG-3ʹ. Experiments using the *Dyrk1a* heterozygous mutant model confirmed the presence of the mutated allele with the primers 5ʹ-ATTCGCAGCGCATCGCCTTCTATCGCC-3ʹ and CGTGATGAGCCCTTACCTATG-3ʹ using a previously described protocol (Fotaki et al., 2002). Presence of the rtTA allele was determined by PCR using primers 5ʹ-CTGCTGTCCATTCCTTATTC-3ʹ, 5ʹ-CGAAACTCTGGTTGACATG-3ʹ and 5ʹ-TGCCTATCATGTTGTCAAA-3ʹ to produce the carrier 330 bp and wild-type 363 bp bands (Paterson et al., 2015; Takiguchi et al., 2013). Presence of the tetO-cre allele in test mice was determined by PCR using the Cre primers 5ʹ-ATTCTCCCACCGTCAGTACG-3ʹ and 5ʹ-CGTTTTCTGAGCATACCTGGA-3ʹ (Chai et al., 2000; Miwa et al., 2009) and the internal positive control primers 5ʹ-CAAATGTTGCTTGTCTGGTG-3ʹ and 5ʹ-GTCAGTCGAGTGCACAGTTT-3ʹ (Deitz and Roper, 2011) to produce the carrier 475 bp and wild-type 200 bp bands when resolved on a 1.5% agarose gel.

### Cre activation

Doxycycline was administered in chow (Envigo, Teklad Custom Diet, TD.120769 – 998.975 g/kg 2018 Teklad Global 18% Protein Rodent Diet, 0.625 g/kg doxycycline hyclate and 0.4 g/kg blue food color), delivering an average daily dose of 2-3 mg doxycycline per 4-5 g chow consumed per day. Doxycycline chow was introduced at the time of weaning ∼ P21. Excision of the floxed region spanning exons 5 and 6 on one allele of *Dyrk1a* in Ts65Dn,*Dyrk1a*^fl/wt^,tetO-cre^+^,rtTA^+/−^ mice that had received doxycycline feed was verified with PCR using the primers 5ʹ-ACCTGGAGAAGAGGGCAAGA-3ʹ and 5ʹ-GCCACTGTGTGAGGAGTCTT-3ʹ on tail DNA taken at P6 (before doxycycline administration) and P30 (after doxycycline administration) (Thomas et al., 2021) (Fig. S1A). After doxycycline administration, the excision was also confirmed in the following tissues: muscle, thymus, heart, lung, liver, spleen and kidney (Fig. S1B).

### Micro-CT and analysis

Scans and analysis were performed on right femurs as described (Sloan et al., 2023) using a SkyScan 1172 high-resolution micro-computed tomography (micro-CT) system (Bruker, Kontich, Belgium). Flat-field corrections occurred prior to scanning. Hydroxyapatite phantoms (0.25 and 0.75 g/cm^3^ calcium hydroxyapatite) were scanned once per week of scanning. Scanning parameters were as follows: 60 kV, 12 µm resolution, 885 ms integration time, 0.7° angular increment and frame averaging of 4. The entire length of the femurs from P12, P15, P18, P24 and P27 were scanned, but owing to size constraints, samples from P30 and P42 were scanned from the distal condyles of the femur to at least the third trochanter. Trabecular microarchitecture was analyzed by identifying a trabecular region of interest beginning at the proximal end of the distal growth plate and extending proximally, based on 10% of the bone length, from the proximal end of the distal growth plate to the widest region of the third trochanter. This analysis was performed using the SkyScan CT Analyzer and a custom MATLAB code to exclude the outer cortical bone, using a lower greyscale threshold of 45 (P12 mice) or 55 (all other ages) and an upper greyscale threshold of 255, as described in previous publications (Goodlett et al., 2020; Stringer et al., 2017a; Thomas et al., 2020; Sloan et al., 2023). The cortical region of interest was defined as a region of seven transverse slices at 60% of the overall length of the bone from the proximal end of the distal growth plate, and cortical geometry was measured as previously described using MATLAB (Goodlett et al., 2020; Stringer et al., 2017a; Thomas et al., 2020; Sloan et al., 2023). Skeletal geometry measures are expressed as standardized nomenclature, symbols and units for bone micro-CT according to American Society for Bone and Mineral Research guidelines (Bouxsein et al., 2010).

### Femoral RNA isolation

RNA was isolated from the cortical midshaft left femora using TRIzol-chloroform as summarized below. After thawing to room temperature (RT), the proximal and distal ends of femora were excised using a razor blade. The cortical midshaft was placed in a modified 200 μl pipette tip and located inside a 1.5 ml microcentrifuge tube. Femora were centrifuged for 10 min at 16,100 ***g*** (Eppendorf 5702 centrifuge with F45-24-11 rotor) to remove the marrow. Femora were then placed into a pre-chilled mortar containing liquid nitrogen and pulverized into a fine powder. The bone powder was then scraped into a nuclease-free 1.5 ml microcentrifuge tube and combined with 50 μl TRIzol before homogenizing with a motorized hand homogenizer for 60 s. 150 μl TRIzol was added and homogenized again. Another 300 μl of TRIzol was added, and the sample was vortexed and then centrifuged at 4°C for 10 min at 9300 ***g***. The supernatant was transferred to another 1.5 ml tube and the process was repeated by adding 50 μl, 150 μl and 300 μl of TRIzol to the pellet after intermittent mixing with a hand homogenizer and vortexing. After the supernatant was incubated at RT for 5 min, chloroform was added to the supernatant and shaken for 15 s. The aqueous and organic layer were allowed to separate by incubation at RT for 5 min. The samples were then centrifuged at 4°C for 10 min at 9300 ***g***, after which the aqueous layer (top) was transferred to a new tube. RNA was precipitated by adding 800 μl of 100% isopropanol. After a 5 min incubation at RT, samples were centrifuged at 4°C for 15 min at 16,100 ***g***. The supernatant was discarded, and 1 ml of 75% ethanol was added to each sample and shaken for 15 s, after which samples were centrifuged at 4°C for 15 min at 16,100 ***g***. The supernatant was discarded and centrifuged again at 4°C for 1 min at 16,100 ***g*** to consolidate the supernatant. The remaining supernatant was aspirated and tubes were left open for 5 min for ethanol evaporation. RNA was resuspended with 20 μl of high-performance liquid chromatography-grade water, the concentration was quantified using a NanoDrop 2000 (Thermo Fisher Scientific), and RNA was stored at −80°C for future use.

### cDNA conversion and gene expression analysis by qPCR

Isolated and quantified RNA samples were converted to cDNA with a final volume of 20 μl using TaqMan Reverse Transcription reagents following the recommended protocol, except for doubling the time of the elongation step. The resulting product was diluted 1:5 with sterile Milli-Q (Millipore Sigma) water and stored at −20°C until analysis. Real-time qPCR was performed using an Applied Biosystems 7500 Real-Time PCR System on 96-well plates. Reagents consisted of TaqMan Gene Expression Master Mix (Roche Diagnostics), TaqMan control probes for *Rn18s* (Roche Diagnostics, Mm03928990_g1), two probes for different regions of the *Dyrk1a* transcript – the first probe targeting a region spanning *Dyrk1a* exons 5 and 6 (Mm00432929_m1) and the second targeting *Dyrk1a* exons 10 and 11 (Mm00432934_m1), and probes for *Bglap* (Mm03413826_m1), *Runx2* (Mm00501584_m1), *Rbl2* (Mm01242468_m1) and *Alpl* (Mm00475834_m1).

The normalized reporter (Rn) was defined as the difference between the reporter signal, FAM, and the quencher, ROX. The difference between the Rn of a given cycle and the Rn of the first cycle was defined as ΔRn. The ΔRn threshold was set at 0.1. The cycle threshold (CT) of 0.100 was chosen because it was a positive threshold that captured the exponential growth phase for all target genes. The difference between the gene of interest *Dyrk1a* and the housekeeping gene *Rn18s* in each sample (CT*_Dyrk1a_* – CT*_Rn18s_*) was defined as ΔCT. As *Rn18s* expression is conserved between genotypes, the ΔCT calculation produced a value that would be different between genotypes if *Dyrk1a* was overexpressed. To obtain relative gene expression, each individual sample measure was normalized using the 2^−ΔCT^ method (2^−ΔCT^=ΔCT_Ts65Dn_ – ΔCT_WT_). To determine the fold change of *Dyrk1a* expression between genotypes, each 2^−ΔCT^ was divided by the mean euploid 2^−ΔCT^ (2^−ΔCT^_WT_/2^−ΔCT^_Ts65Dn_) and the average of the relative fold change was used to quantify *Dyrk1a* gene expression. The resulting values can only be positive; overexpression of a gene would be indicated by a value greater than 1, whereas under-expression of a gene would be indicated by a value between 0 and 1.

### Treatment of CX-4945 in Ts65Dn,*Dyrk1a*^fl/wt^ mice

Ts65Dn,*Dyrk1a*^fl/wt^ and control male mice were treated with 75 mg/kg/day CX-4945 (Silmitasertib, SelleckChem) as a 10% DMSO:90% PBS suspension delivered via oral gavage. A 250 mM solution with 24.5 mg CX-4945 and 280 μl DMSO was gently heated in a 37°C water bath or heat block. The working suspension was made daily by diluting the stock solution to 25 mM with PBS to make a suspension (10% total DMSO) and kept in a 37°C heat block until administered to weaned (P21) to P29 mice. Control animals received a vehicle treatment composed of 10% DMSO and 90% PBS.

### Statistical analyses

Skeletal parameters from micro-CT were analyzed using IBM SPSS Statistics (v29.0.1.0). Normality was assessed using Shapiro–Wilk's test (α=0.05) and non-normal data were logarithmically transformed and tested again. Homogeneity of variance was assessed using Levene's test (α=0.05). *P-*values generated using one- and two-way ANOVA and two-tailed unpaired *t*-tests were adjusted using the Benjamini–Hochberg method of false rate discovery (FDR) correction separately for trabecular and cortical parameters. Significance was determined as an adjusted *P*≤0.05. In cases where Levene's test was significant, indicating violation of homogeneity of variance, Welch's one-way ANOVA was used to confirm significance (α=0.05), and a parameter was reported as significant if both the FDR-adjusted ANOVA and Welch's ANOVA produced a significant *P*-value. For the development of the Ts65Dn skeletal phenotype from the P12-P42 experiment, two-way ANOVAs among genotype, age and their interaction were performed with Tukey's (non-significant Levene's test) or Games–Howell (significant Levene's test) post hoc analysis in cases of a significant age effect (no interactions were detected). Additionally, two-tailed unpaired *t*-tests were performed between the two genotypes for each age. For the CX-4945 treatment experiment, two-way ANOVAs among genotype, treatment and their interaction were performed. Additionally, a repeated-measures ANOVA was performed for body weight, using genotype and treatment as between-subject factors and postnatal day as the within-subject factor. Sphericity was assessed using Mauchly's test, and Greenhouse–Geisser test was used for within-subject effect due to significant Mauchly's test. Pairwise comparisons with a Bonferroni adjustment were used in cases where the within-subject effect or interactions involving the within-subject effect were significant. For the temporal reduction of *Dyrk1a* experiment, one-way ANOVAs were performed between the four genotype groups with Tukey's or Games–Howell post hoc analysis when significant. With the P30 and P36 germline reduction of *Dyrk1a* copy number experiments, two-way ANOVAs were performed among genotype, sex and their interaction with Tukey's or Games–Howell post hoc analysis in cases of a significant genotype effect and/or interaction. Additionally, one-way ANOVAs were performed among the four genotype groups for each sex with Tukey's or Games–Howell post hoc analysis when significant. Significance for qPCR-based *Dyrk1a* and other osteogenic gene expression measures was determined by unpaired one-tailed *t*-tests. Normality was not violated at any age (GraphPad Prism 9.0.1).

## Supplementary Material

10.1242/dmm.050914_sup1 Supplementary information
