# Supplementary material for: Sex-specific trisomic Dyrk1a-related skeletal phenotypes during development in a Down syndrome model
Source: Dis Model Mech. 2024 Sep 23;17(9):dmm050914. doi: 10.1242/dmm.050914 (PMC11449447; doi:10.1242/dmm.050914)
Supplement: Supplementary information [file dmm-17-050914-s1.pdf]

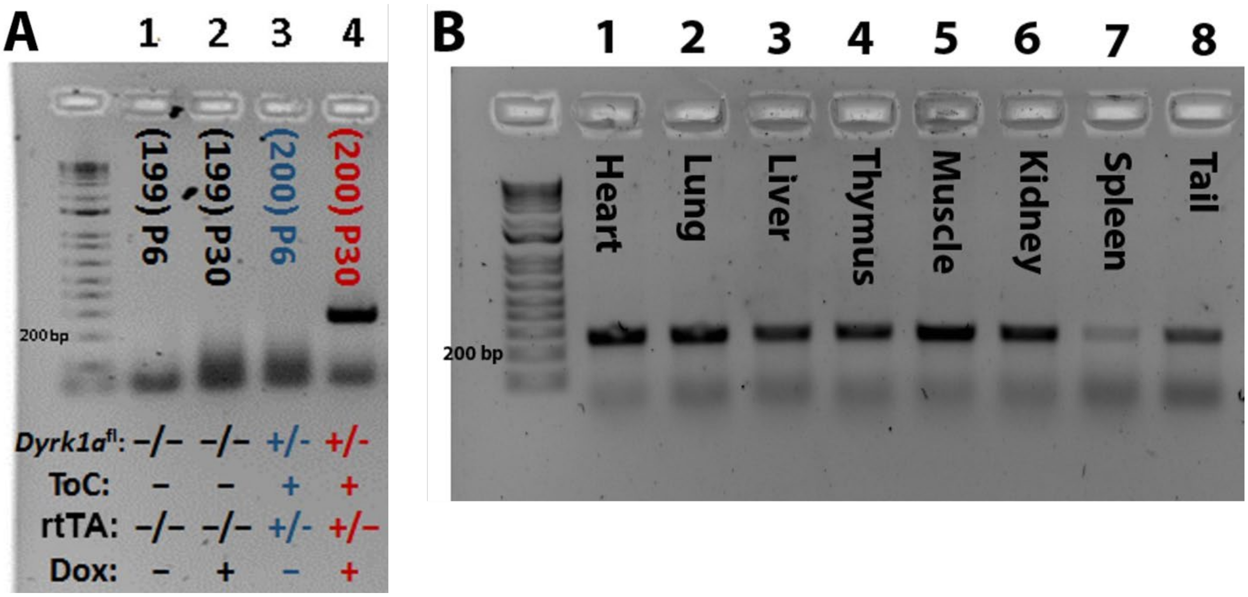

Fig. S1. PCR verification of temporal conditional reduction of *Dyrk1a* exon 5-6. A) *Dyrk1a* excision PCR from tail DNA taken at postnatal day 6 (P6) (before doxycycline administration) and P30 (after doxycycline administration). Animal 199 (lanes 1 & 2) lacks all components for the TET-on system. Animal 200 (lanes 3 & 4) has all components, and a truncated *Dyrk1a* amplicon (214 bp) can be seen at P30 (lane 4). B) *Dyrk1a* excision PCR from multiple tissues of an animal post-doxycycline administration. All tissues sampled were from animals containing a truncated *Dyrk1a* amplicon.

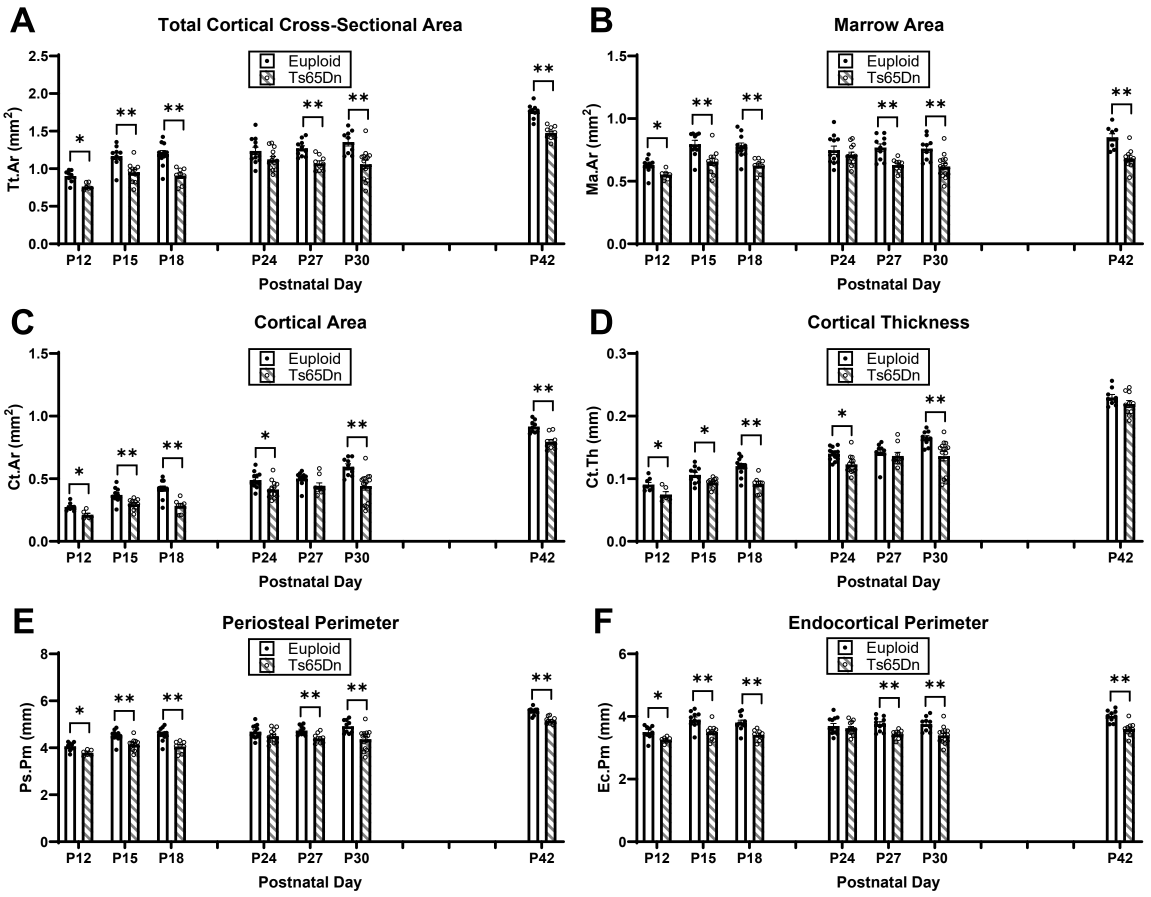

Fig. S2. Cortical analysis of male Ts65Dn femurs from postnatal day (P)12-42. Remaining cortical parameters from µCT analysis. Data are mean ± SEM. Significance determined through two-tail t-test with FDR adjustment. (*) indicates p ≤ 0.05, (**) indicates p ≤ 0.01.

Disease Models & Mechanisms • Supplementary information

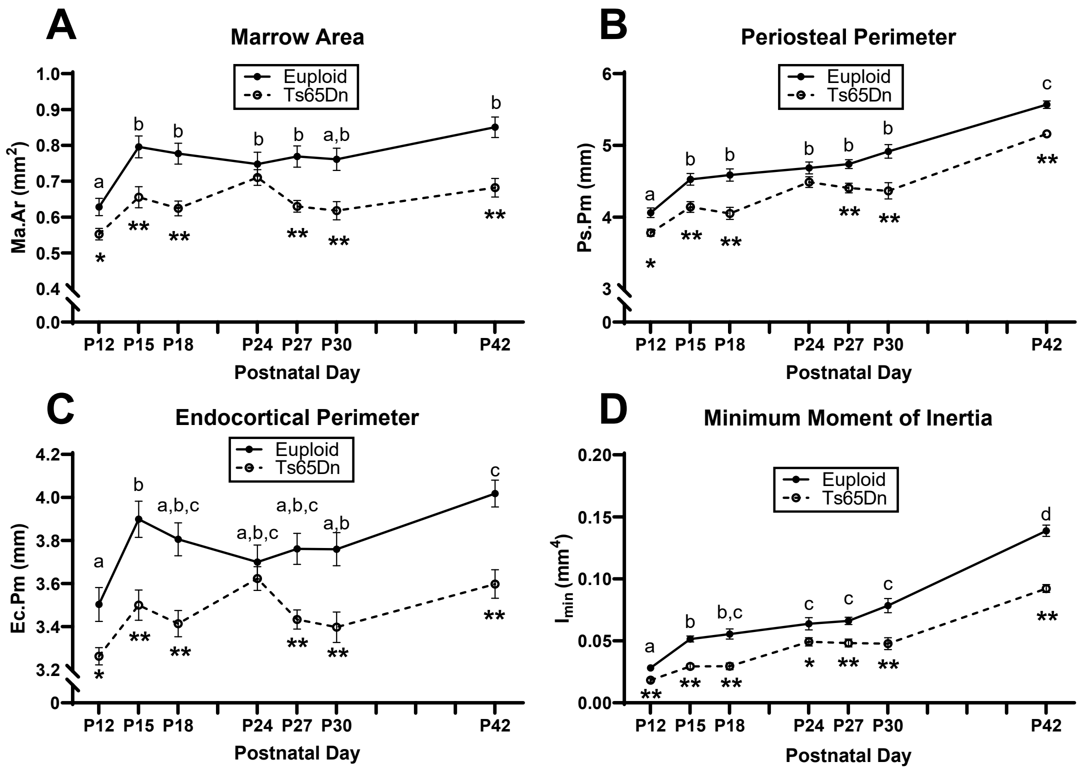

Fig. S3. Cross-sectional growth trajectory of remaining cortical bone parameters in male euploid and Ts65Dn femurs from postnatal day (P)12-42. Data are mean ± SEM. Letters (a,b,c,d) indicate significant differences between ages by two-way ANOVA and Tukey/Games-Howell *post hoc* analysis (age effect); ages with the same letter are not significantly different from one another. (*) indicates adjusted $p \leq 0.05$ by two-tail t-test between euploid and Ts65Dn mice at the given age. (**) indicates adjusted $p \leq 0.01$ by two-tail t-test between euploid and Ts65Dn mice at the given age. See Fig. 1 for group numbers.

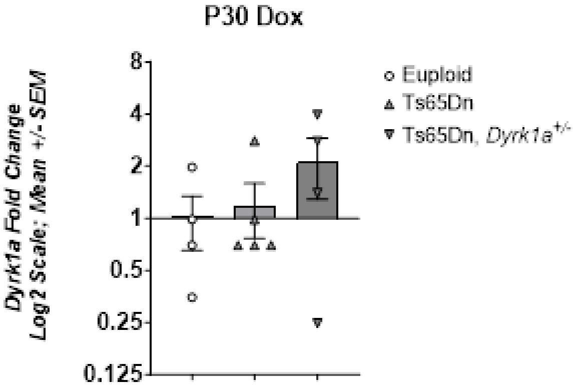

Fig. S4. Expression of *Dyrk1a* in male offspring from (Ts65Dn,*Dyrk1a*$^{fl/wt}$ × rtTA^{+}, TetOCre^{+}) matings given doxycycline (Dox).

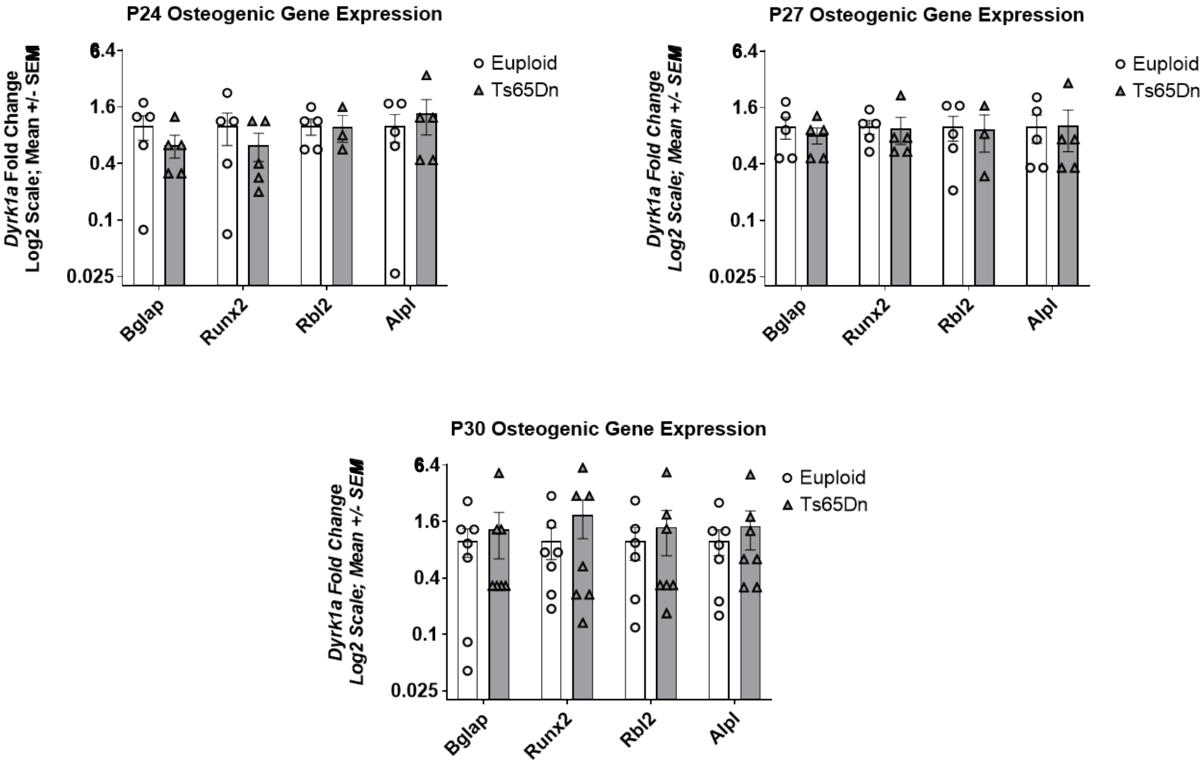

Fig. S5. Osteogenic gene expression at P24, P27, and P30.

Table S1. Significant *p* values from two-way ANOVA (genotype x age) for male P12-P42 Ts65Dn trabecular and cortical parameters. All effect and interaction *p* values reported are adjusted using the Benjamini-Hochberg method. Welch's t-test was performed to confirm ANOVA results when a significant Levene's test occurred, indicating unequal variances between groups, and these *p* values are unadjusted. If Welch's t-test encountered a non-significant result when ANOVA generated a significant result, the non-significant result was reported. ǂ indicates the data was log-transformed for statistical analysis due to non-normal distribution. NS indicates *p* value was not significant. N/A indicates Welch's t-test was not performed because Levene's test was not significant. See Fig. 1 for group numbers.

	**BMD**	**BV/TV**	**Tb.Th**	**Tb.Sp**	**Tb.N**
Genotype Effect	0.003	0.003	0.008	0.003	0.003
Welch's t-test	NS	NS	NS	0.007	0.014
Age Effect	0.003	0.003	0.003	0.003	0.003
Welch's t-test	<0.001	<0.001	<0.001	<0.001	<0.001
Interaction	NS	NS	NS	NS	NS

	**Tt.Ar**^{ǂ}	**Ma.Ar**^{ǂ}	**Ct.Ar**^{ǂ}	**Ct.Th**	**Ps.Pm**	**Ec.Pm**	$I_{max}^{ǂ}$	$I_{min}^{ǂ}$	**Ct.TMD**
Genotype Effect	0.003	0.003	0.003	0.003	0.003	0.003	0.003	0.003	0.003
Welch's t-test	<0.001	N/A	0.004	NS	<0.001	N/A	<0.001	<0.001	NS
Age Effect	0.003	0.003	0.003	0.003	0.003	0.003	0.003	0.003	0.003
Welch's t-test	<0.001	N/A	<0.001	<0.001	<0.001	N/A	<0.001	<0.001	<0.001
Interaction	NS	NS	NS	NS	NS	NS	NS	NS	NS

Table S2. Significant *p* values from two-way ANOVA (genotype x treatment) for male P30 CX-4945-treated Ts65Dn trabecular and cortical parameters and body weight. All effect and interaction *p* values reported are adjusted using the Benjamini-Hochberg method. ǂ indicates the data was log-transformed for statistical analysis due to non-normal distribution. NS indicates *p* value was not significant. Vehicle-treated euploid (n = 10), CX-4945-treated euploid (n = 13), vehicle-treated Ts65Dn (n = 13), CX-4945-treated Ts65Dn (n = 8).

	BMD	BV/TV	Tb.Th	Tb.Spǂ	Tb.N	Average Weight	Weight Gain
Genotype Effect	0.008	0.008	NS	0.008	0.008	<0.001	NS
Treatment Effect	NS	NS	NS	NS	NS	NS	NS
Interaction	NS	NS	NS	NS	NS	NS	NS

	Tt.Ar	Ma.Ar	Ct.Ar	Ct.Th	Ps.Pm	Ec.Pm	I_{max}	I_{min}	Ct.TMD
Genotype Effect	0.005	0.005	0.005	0.005	0.005	0.005	0.005	0.005	0.005
Treatment Effect	NS	NS	NS	NS	NS	NS	NS	NS	NS
Interaction	NS	NS	NS	NS	NS	NS	NS	NS	NS

Disease Models & Mechanisms • Supplementary information

Table S3. Average and standard error of the mean (SEM) of µCT parameters at postnatal day (P) 30 and daily body weight at each postnatal day (P) from start of treatment to day of euthanasia for male Ts65Dn and euploid mice treated with vehicle (90%PBS:10%DMSO) or CX-4945

	BMD	BV/TV	Tb.Th	Tb.Sp	Tb.N
Euploid + Vehicle	0.199 ± 0.010	26.07 ± 2.07	0.069 ± 0.001	0.160 ± 0.010	3.78 ± 0.274
Euploid + CX-4945	0.222 ± 0.005	30.16 ± 0.93	0.070 ± 0.0007	0.144 ± 0.005	4.30 ± 0.129
Ts65Dn + Vehicle	0.181 ± 0.008	23.05 ± 1.41	0.069 ± 0.001	0.187 ± 0.010	3.30 ± 0.183
Ts65Dn + CX-4945	0.180 ± 0.012	22.85 ± 2.07	0.068 ± 0.002	0.177 ± 0.011	3.34 ± 0.255

	Tt.Ar	Ma.Ar	Ct.Ar	Ct.Th	Ps.Pm	Ec.Pm	I_{max}	I_{min}	Ct.TMD
Euploid + Vehicle	1.39 ± 0.040	0.772 ± 0.021	0.618 ± 0.022	0.170 ± 0.004	4.94 ± 0.064	3.78 ± 0.048	0.135 ± 0.008	0.085 ± 0.005	1.01 ± 0.008
Euploid + CX-4945	1.40 ± 0.035	0.770 ± 0.028	0.627 ± 0.014	0.172 ± 0.003	4.97 ± 0.057	3.78 ± 0.063	0.140 ± 0.006	0.084 ± 0.004	1.01 ± 0.005
Ts65Dn + Vehicle	1.20 ± 0.049	0.686 ± 0.026	0.516 ± 0.025	0.151 ± 0.005	4.64 ± 0.096	3.57 ± 0.069	0.102 ± 0.009	0.062 ± 0.004	0.974 ± 0.008
Ts65Dn + CX-4945	1.14 ± 0.058	0.638 ± 0.033	0.505 ± 0.026	0.153 ± 0.005	4.52 ± 0.110	3.44 ± 0.086	0.095 ± 0.011	0.056 ± 0.005	0.984 ± 0.005

Weight:	P21	P22	P23	P24	P25	P26	P27	P28	P29	P30
Euploid + Vehicle	11.58 ± 0.64	12.03 ± 0.64	12.66 ± 0.60	13.52 ± 0.61	14.44 ± 0.64	15.38 ± 0.67	16.39 ± 0.67	17.16 ± 0.69	18.25 ± 0.68	18.58 ± 0.58
Euploid + CX-4945	11.85 ± 0.38	12.62 ± 0.43	13.34 ± 0.45	14.12 ± 0.48	14.98 ± 0.53	15.83 ± 0.51	16.70 ± 0.53	17.36 ± 0.50	17.98 ± 0.50	18.69 ± 0.49
Ts65Dn + Vehicle	8.52 ± 0.58	8.82 ± 0.58	9.44 ± 0.58	10.28 ± 0.58	11.05 ± 0.63	12.02 ± 0.69	12.85 ± 0.66	13.82 ± 0.70	14.88 ± 0.74	15.72 ± 0.77
Ts65Dn + CX-4945	8.36 ± 0.75	9.06 ± 0.82	9.76 ± 0.89	10.53 ± 0.92	11.38 ± 1.01	12.24 ± 1.07	12.88 ± 1.12	13.49 ± 1.17	14.04 ± 1.20	14.49 ± 1.23

Table S4. Significant *p* values from one-way ANOVA (genotype) for male P30 Ts65Dn,*Dyrk1a*$^{fl/+}$,rtTA^{+},TetOCre^{+} (Ts65Dn × *Dyrk1a*$^{+/dox\text{-}cre}$ offspring) trabecular and cortical parameters. All effect and interaction *p* values reported are adjusted using the Benjamini-Hochberg method. Welch's t-test was performed to confirm ANOVA results when a significant Levene's test occurred, indicating unequal variances between groups, and these p values are unadjusted. All *p* values from *post hoc* analyses are unadjusted. ǂ indicates the data was log-transformed for statistical analysis due to non-normal distribution. NS indicates *p* value was not significant. N/A indicates Welch's t-test was not performed because Levene's test was not significant or *post hoc* analysis was not performed because one-way ANOVA was not significant. GH stands for Games-Howell *post hoc* analysis. Euploid (n = 10), euploid,*Dyrk1a*$^{+/dox\text{-}cre}$ (n = 7), Ts65Dn (n = 11), Ts65Dn,*Dyrk1a*$^{+/+/dox\text{-}cre}$ (n = 4).

	BMD	BV/TVǂ	Tb.Th	Tb.Sp	Tb.N
Genotype	0.003	0.003	NS	0.003	0.003
Welch's t-test	N/A	N/A	N/A	0.001	N/A
Post Hoc	***Tukey***	***Tukey***	***N/A***	***GH***	***Tukey***
Eu v. Eu,Dyrk1a$^{+/dox\text{-}cre}$	NS	NS	N/A	NS	NS
Eu v. Ts	0.027	0.022	N/A	NS	NS
Eu v. Ts,Dyrk1a$^{+/+/dox\text{-}cre}$	0.022	0.009	N/A	0.042	0.023
Ts v. Ts,Dyrk1a$^{+/+/dox\text{-}cre}$	NS	NS	N/A	NS	NS
Eu,Dyrk1a$^{+/dox\text{-}cre}$ *v. Ts*	0.019	0.008	N/A	0.002	0.001
Eu,Dyrk1a$^{+/dox\text{-}cre}$ *v. Ts,Dyrk1a*$^{+/+/dox\text{-}cre}$	0.015	0.004	N/A	0.009	0.001

	Tt.Ar	Ma.Ar	Ct.Ar	Ct.Th	Ps.Pm	Ec.Pm	I_{max}	I_{min}	TMD
Genotype	0.002	0.002	0.002	0.002	0.002	0.002	0.002	0.002	0.002
Welch's t-test	0.001	0.001	N/A	N/A	N/A	0.001	0.001	0.001	N/A
Post Hoc	***GH***	***GH***	***Tukey***	***Tukey***	***Tukey***	***GH***	***GH***	***GH***	***Tukey***
Eu v. Eu,Dyrk1a$^{+/dox\text{-}cre}$	NS	NS	NS	NS	NS	NS	NS	NS	NS
Eu v. Ts	0.004	0.022	0.001	0.001	0.001	0.010	0.006	0.004	0.001
Eu v. Ts,Dyrk1a$^{+/+/dox\text{-}cre}$	0.009	0.027	0.001	0.001	0.003	0.013	0.007	0.008	0.032
Ts v. Ts,Dyrk1a$^{+/+/dox\text{-}cre}$	NS	NS	NS	NS	NS	NS	NS	NS	NS
Eu,Dyrk1a$^{+/dox\text{-}cre}$ *v. Ts*	0.001	0.001	0.001	0.004	0.002	0.001	0.001	0.001	0.001
Eu,Dyrk1a$^{+/dox\text{-}cre}$ *v. Ts,Dyrk1a*$^{+/+/dox\text{-}cre}$	0.013	0.001	0.005	0.007	0.017	0.003	0.009	0.008	0.002

Disease Models & Mechanisms • Supplementary information

Table S5. Average and standard error of the mean (SEM) of µCT parameters and body weight for temporal reduction of *Dyrk1a* copy number in male Ts65Dn mice at P30.

	BMD	BV/TV	Tb.Th	Tb.Sp	Tb.N	Body Weight
Euploid	0.203 ± 0.016	26.98 ± 3.03	0.069 ± 0.004	0.166 ± 0.010	3.83 ± 0.232	18.67 ± 0.81
***Eu,Dyrk1a*$^{+/dox-cre}$**	0.211 ± 0.012	28.79 ± 2.23	0.063 ± 0.002	0.142 ± 0.005	4.53 ± 0.234	18.04 ± 0.60
Ts65Dn	0.152 ± 0.010	18.27 ± 1.70	0.060 ± 0.001	0.212 ± 0.014	3.02 ± 0.242	13.02 ± 0.86
***Ts,Dyrk1a*$^{+/+/dox-cre}$**	0.132 ± 0.013	14.73 ± 1.79	0.058 ± 0.002	0.213 ± 0.011	2.52 ± 0.272	12.15 ± 0.78

	Tt.Ar	Ma.Ar	Ct.Ar	Ct.Th	Ps.Pm	Ec.Pm	I_{max}	I_{min}	Ct.TMD
Euploid	1.46 ± 0.075	0.812 ± 0.047	0.646 ± 0.031	0.173 ± 0.005	5.10 ± 0.122	3.90 ± 0.104	0.155 ± 0.016	0.092 ± 0.009	1.02 0.007
***Eu,Dyrk1a*$^{+/dox-cre}$**	1.42 ± 0.033	0.801 ± 0.011	0.620 ± 0.026	0.168 ± 0.006	5.01 ± 0.057	3.86 ± 0.025	0.141 ± 0.009	0.086 ± 0.005	1.04 ± 0.005
Ts65Dn	1.09 ± 0.039	0.636 ± 0.023	0.458 ± 0.019	0.140 ± 0.004	4.46 ± 0.078	3.45 ± 0.062	0.082 ± 0.007	0.050 ± 0.003	0.980 ± 0.006
***Ts,Dyrk1a*$^{+/+/dox-cre}$**	1.08 ± 0.056	0.646 ± 0.015	0.440 ± 0.042	0.134 ± 0.011	4.44 ± 0.125	3.47 ± 0.047	0.076 ± 0.011	0.049 ± 0.006	0.985 ± 0.012

Table S6. Comparison of P30 vehicle (10%DMSO:90%PBS)-treated and untreated euploid and Ts65Dn mice. Data are average ± SEM.

	BMD (g/cm^3)	BV/TV (%)	Tb.Th (mm)	Tb.Sp (mm)	Tb.N (1/mm)	Body Weight (g)
P30 untreated euploid	0.181 ± 0.012	23.94 ± 2.44	0.065 ± 0.001	0.171 ± 0.014	3.65 ± 0.320	19.63 ± 0.99
P30 vehicle-treated euploid	0.199 ± 0.010	26.07 ± 2.07	0.069 ± 0.001	0.160 ± 0.010	3.78 ± 0.274	18.58 ± 0.58
P30 untreated Ts65Dn	0.132 ± 0.013	15.32 ± 2.26	0.062 ± 0.002	0.245 ± 0.022	2.36 ± 0.312	12.74 ± 1.16
P30 vehicle-treated Ts65Dn	0.181 ± 0.008	23.05 ± 1.41	0.069 ± 0.001	0.187 ± 0.010	3.30 ± 0.183	15.72 ± 0.77

	Tt.Ar (mm^2)	Ma.Ar (mm^2)	Ct.Ar (mm^2)	Ct.Th (mm)	Ps.Pm (mm)	Ec.Pm (mm)	I_{max} (mm^4)	I_{min} (mm^4)	Ct.TMD (g/cm^3)
P30 untreated euploid	1.36 ± 0.053	0.761 ± 0.031	0.595 ± 0.024	0.165 ± 0.004	4.92 ± 0.094	3.76 ± 0.077	0.131 ± 0.011	0.078 ± 0.006	1.01 ± 0.004
P30 vehicle-treated euploid	1.39 ± 0.040	0.772 ± 0.021	0.618 ± 0.022	0.170 ± 0.004	4.94 ± 0.064	3.78 ± 0.048	0.135 ± 0.008	0.085 ± 0.005	1.01 ± 0.008
P30 untreated Ts65Dn	1.06 ± 0.055	0.618 ± 0.025	0.442 ± 0.032	0.136 ± 0.007	4.37 ± 0.114	3.40 ± 0.071	0.079 ± 0.010	0.060 ± 0.005	0.964 ± 0.012
P30 vehicle-treated Ts65Dn	1.20 ± 0.049	0.686 ± 0.026	0.516 ± 0.025	0.151 ± 0.005	4.64 ± 0.096	3.57 ± 0.069	0.102 ± 0.009	0.062 ± 0.004	0.974 ± 0.008

Disease Models & Mechanisms • Supplementary information
